# Proline-rich tyrosine kinase 2 mediates transforming growth factor-beta-induced hepatic stellate cell activation and liver fibrosis

**DOI:** 10.1038/s41598-020-78056-0

**Published:** 2020-12-03

**Authors:** Jonghwa Kim, Wonseok Kang, So Hee Kang, Su Hyun Park, Ji Young Kim, Sera Yang, Sang Yun Ha, Yong-Han Paik

**Affiliations:** 1 Department of Medicine, Samsung Medical Center, Sungkyunkwan University School of Medicine, 81 Irwon-Ro Gangnam-gu, Seoul, 06351 Korea; 2 Department of Health Sciences and Technology, SAIHST, Sungkyunkwan University, Seoul, Korea; 3 Department of Pathology, Samsung Medical Center, Sungkyunkwan University School of Medicine, Seoul, Korea

**Keywords:** Hepatic stellate cells, Focal adhesion

## Abstract

Hepatic fibrogenesis is characterized by activation of hepatic stellate cells (HSCs) and accumulation of extracellular matrix (ECM). The impact of ECM on TGF-β-mediated fibrogenic signaling pathway in HSCs has remained obscure. We studied the role of non-receptor tyrosine kinase focal adhesion kinase (FAK) family members in TGF-β-signaling in HSCs. We used a CCl_4_-induced liver fibrosis mice model to evaluate the effect of FAK family kinase inhibitors on liver fibrosis. RT-PCR and Western blot were used to measure the expression of its target genes; α-SMA, collagen, Nox4, TGF-β1, Smad7, and CTGF. Pharmacological inhibitors, siRNA-mediated knock-down, and plasmid-based overexpression were adopted to modulate the function and the expression level of proteins. Association of PYK2 activation with liver fibrosis was confirmed in liver samples from CCl_4_-treated mice and patients with significant fibrosis or cirrhosis. TGF-β treatment up-regulated expression of α-SMA, type I collagen, NOX4, CTGF, TGF-β1, and Smad7 in LX-2 cells. Inhibition of FAK family members suppressed TGF-β-mediated fibrogenic signaling. SiRNA experiments demonstrated that TGF-β1 and Smad7 were upregulated via Smad-dependent pathway through FAK activation. In addition, CTGF induction was Smad-independent and PYK2-dependent. Furthermore, RhoA activation was essential for TGF-β-mediated CTGF induction, evidenced by using ROCK inhibitor and dominant negative RhoA expression. We identified that TGF-β1-induced activation of PYK2-Src-RhoA triad leads to YAP/TAZ activation for CTGF induction in liver fibrosis. These findings provide new insights into the role of focal adhesion molecules in liver fibrogenesis, and targeting PYK2 may be an attractive target for developing novel therapeutic strategies for the treatment of liver fibrosis.

## Introduction

Chronic liver injury leads to liver fibrosis, cirrhosis and eventually organ failure. Liver fibrosis is characterized by dysregulated production of extracellular matrix (ECM) by activated hepatic stellate cells (HSCs)^1,2^. Several signal pathways have been studied extensively in the context of fibrogenesis, including transforming growth factor (TGF)-β and Hippo pathway^1,3–8^.

TGF-β and connective tissue growth factor (CTGF) are the primary factors driving the excessive production of ECM by activated HSCs during the wound-healing response^9–11^. The canonical TGF-β signaling pathway is transmitted through phosphorylation of Smad proteins which translocates to the nucleus and induces transcription of target genes, such as *TGFB1*, *SMAD7*, and *CTGF*^12^. In contrast, the non-canonical TGF-β signaling pathway activates PI3K, ROCK, ERK, JNK, and MAPK via AKT, RhoA, Ras, and TAK1, respectively, in the absence of Smad phosphorylation^12^. Although CTGF is regarded as a master pro-fibrogenic mediator in TGF-β-mediated ECM expression, the signal transduction mechanism by which CTGF exerts remains unclear.

Several in vivo and in vitro evidences demonstrated that YAP drives HSC activation in response to extracellular stiffness; the stiffness-driven activation is enhanced in the presence of TGF-β1, and requires adhesion to matrix proteins^4,8^. Other than hepatic fibrosis, YAP activation has also been observed in renal and pulmonary fibrosis, where knockdown of YAP suppresses matrix deposition and proliferation of their fibroblasts^13–15^. Notably, observed commonly in these three organs is a positive feedback loop, where YAP activation leads to ECM deposition via fibroblast cells and increased extracellular stiffness by ECM deposition in turn results in further sustainable activation of YAP. However, the precise mechanisms of crossing mechanical signals to intracellular signaling pathway to modulate YAP are still unclear.

Cells interact with the ECM components primarily at focal adhesions through surface integrin heterodimers, with its extracellular domains interacting with ECM components and its cytosolic C-terminal domains interacting with various cellular signaling components, including actin, and focal adhesion proteins^16^. Integrins are able to transmit extracellular mechanical signals across the membrane, via “inside-out” and “outside-in” signaling pathways^17^. Recently, integrin-α_11_β_1_ was demonstrated to modulate HSC activation via Hippo/YAP pathway and promote liver fibrosis^18^. However, the more molecular details of signal transmissions through integrin to YAP modulation for liver fibrosis are still vague.

Focal adhesion kinase (FAK) family of non-receptor tyrosine kinases is composed of FAK and proline-rich tyrosine kinases 2 (PYK2)^19,20^. While FAK is the best studied and representative focal adhesion proteins playing roles in integrin signaling pathways for various cellular processes, such as migration and cell proliferation, PYK2 is known to play redundant roles because of sharing similarity of 40–60% in sequence, similar domain organization, and tyrosine phosphorylation sites, as shown in FAK-knockout cells^19,21–27^. However, there are growing numbers of evidence showing that PYK2 substantially differs from FAK in integrin-mediated regulations, responses to intracellular Ca^2^^+^ concentration, and subcellular localization^19^.

In the present study, we investigated the distinct fibrogenic signaling pathways of two FAK family members, FAK and PYK2, in TGF-β-mediated HSC activation and liver fibrosis.

## Methods

### Animal experiments

Inbred male C57BL/6N mice were purchased from Orient Bio (South Korea). 8-week old mice were divided into four groups; olive oil + vehicle, olive oil + inhibitor, CCl_4_ (Merck, USA) + vehicle, CCl_4_ + inhibitor. CCl_4_ (diluted 1:3 with olive oil (Sigma-Aldrich)) were administrated intraperitoneally at a dose of 0.5 μl/g body weight twice per week as previously described^28,29^. The toxicity of CCl_4_ administration was monitored daily by measuring body weights. Inhibitor of FAK family kinase [PF-431396 in 5% Gelucire 44/14 (Gattefosse, France)] was administrated daily and orally at a dose of 30 mg/kg body weight from the beginning of CCl_4_ administration. After 6 weeks of administration, mice liver tissues were harvested for the subsequent analyses of RT q-PCR, western blots, IHC, and IF. In addition, mice plasmas were collected for measuring levels of aminotransferases (ALT/AST) using Fuji Dri-Chem 3000 and its ALT/AST slide sets (Fuji). All procedures of animal experiments and cell isolation were reviewed and approved by the Institutional Animal Care and Use Committees (IACUC) of Samsung Biomedical Research Institute (SBRI) at Samsung Medical Center (SMC), that abides by the Institute of Laboratory Animal Resources (ILAR) guide and is an accredited facility of the Association for Assessment and Accreditation of Laboratory Animal Care International (AAALAC International). The care and use of animals were carried out in accordance with the relevant guidelines.

### Patient samples

Human liver specimens were provided by SMC Biobank. All the participants involved in the study signed informed consent for the donation to the Biobank and the use of the participants' samples for research purposes under protocols approved by the SMC Institutional Review Board (IRB No. SMC 2015-12-135). All methods were carried out in accordance with relevant guidelines and regulations for human participants.

### Isolation and activation of mouse primary HSCs

Primary quiescent HSCs were isolated from 10 to 20 weeks-old BALB/c mice. as previously described. Briefly, during anesthesia mouse liver perfusion has been performed sequentially with EGTA (Sigma-Aldrich), pronase (Roche), and collagenase (Roche) solution, followed by centrifugation in 8.24% Nycodenz solution (Axis-Shield) or in 17.6% Opti-Prep solution (Axis-Shield). All chemicals to prepare the solutions were purchased from Sigma-Aldrich. After several washing, the isolated HSCs were cultured and activated on plastic tissue culture dishes for 7–10 days. More than 95% purity of the isolated HSCs through the procedure and the activation has been judged by observing the presence of lipid droplets in quiescent states and its disappearance in activated states, as well as immunofluorescent staining of α-SMA in activated mHSCs. The activated HSCs were used without further passaging.

### Cell culture

All cells, including mouse primary HSCs and LX2 (immortalized human HSCs; a gift from Dr. Scott Friedman (Icahn School of Medicine at Mount Sinai, New York, NY)) were maintained in DMEM (Gibco, Thermo Fisher Scientific) with 10% FBS and 100U/ml penicillin–streptomycin (Gibco, Thermo Fisher Scientific). To activate TGF-β1 signaling in vitro, serum-starved cells were stimulated by 5 ng/ml recombinant human TGF-β1 protein (R&D Systems) for 1.5 h in most experiments, with the exception that 10 h or overnight treatment was required to induce expression of α-SMA and type I collagen. All in vitro treatments of inhibitor were performed by pre-treating for 1 h before TGF-β1 stimuli.

### Immunohistochemistry and immunofluorescence analysis

At the time of harvest, the mouse liver tissues were immediately fixed in 10% formalin (Sigma-Aldrich), and after 24 h were subjected to paraffin-embedding and sectioning with 4 μm thickness. For H&E staining, a standard protocol was performed as follows: liver sections were de-paraffinized in oven, hydrated through Xylene (Carlo Ebra) and a series of ethanol (Samchun Chemical, Korea), stained in Harris Hematoxylin (6 min), differentiated in 1% acidic alcohol, counterstained with Eosin (30 s; Sigma), and then subjected to dehydration-Xylene-mounting with VectaMount medium (Vector). For Sirius red staining, the paraffin-embedded sections were de-paraffinized with dry oven and xylene treatment, hydrated through a series of ethanol (100, 95, 90, 80, and 70%), and then stained with picro-sirius red solution (1% Sirius red in picric acid solution; Sigma-Aldrich) for 1 h. After washed with 0.5% acetic acid and dehydrated with ethanol, the sections were cleared in xylene and then mounted. For immunohistochemical (IHC) staining, paraffin-embedded liver sections were dewaxed, rehydrated, and then subjected to antigen retrieval in citrate buffer (pH 6; Dako). After blocking endogenous peroxidase activity with peroxidase-blocking solution (DAKO) and reducing nonspecific background staining with protein block serum-free solution (DAKO), the sections were incubated overnight at 4° C with primary antibodies in wet chamber. After washing three times with PBS-T buffer, slides were incubated with EnVision secondary antibody (DAKO) for 1 h at room temperature. Then, signals were developed by either substrate DAB (DAKO) or AEC (Vector), followed by Mayer’s hematoxylin counterstaining. For double-staining, the first staining was developed by substrate AEC, an alcohol soluble chromogen, and followed by scanning. Then, the slides were stripped by incubating in the stripping buffer (20% SDS, 0.5 M Tris, pH 6.8, 2-mercaptoethanol) for 30 min at 56 °C, de-stained with 100% ethanol for 1 min, and then rehydrated. The second staining were performed by incubating overnight with another primary antibody, followed by secondary antibody. This second staining were developed with substrate DAB and counterstained with Mayer’s hematoxylin. All slides were scanned using Aperio Scanscope AT, and analyzed with the Aperio ImageScope Software (Aperio Technologies).

### RNA isolation and real-time quantitative PCR

With 1 μg of total RNA isolated from frozen cell pellets or mice liver tissues using an RNeasy Mini Kit (Qiagen) and then quantitated at Nano-Drop 200 (Nanodrop), reverse transcription were conducted with High Capacity RNA-to-cDNA kit (Applied Biosystems) according to the manufacturer’s protocol: 37 °C for 60 min, 94 °C for 5 min, and 4 °C for 10 min. RT q-PCR was performed at ABI 7900HT system (Applied Biosystems) with target gene-specific Taqman primer and probe sets from ABI (Supplementary Table 1), for 40 cycles of 15 s at 94 °C and 1 min at 60 °C. By using RQ manager software (Applied Biosystems) for quantification, the relative gene expression values for the target mRNA were normalized to GAPDH or 18 s RNA, calculated using the 2^−ΔΔCT^ method, and expressed as fold increases in comparison with control treatments.

### siRNA-mediated knockdown and overexpression

For siRNA-mediated knockdown, LX2 were seeded in 6 well culture plate with around 80% confluency. At the next day, mixture of siRNA of target genes and Lipofectamine RNAi/MAX reagent (Life Technologies, Thermo Scientific) in Opti-MEM (Life Technologies, Thermo Scientific) were added according to the manufacturer’s instructions. All siRNAs were generated at Bioneer (Korea), of which sequences were obtained from previous reports (Supplementary Table 2). The plasmid transfections were performed using X-tremeGENE HP DNA transfection Reagent (Roche), where 2ug of total plasmid were mixed with 4ul X-tremeGENE reagent in Opti-MEM for each well of 6 well culture plate. In case of doing both knockdown and overexpression in one well, siRNA-mediated knockdown was performed first and then followed by plasmid transfection.

### Immunoblotting analysis

The frozen mouse liver tissues were lysed in T-PER buffer (Thermo Scientific) supplemented with protease inhibitors (Thermo Scientific), phosphatase inhibitors (Thermo Scientific), and stainless steel beads (Qiagen) with TissueLyser II (Qiagen). After clearing by centrifugation, protein concentrations were measured by BCA assay kit (Thermo Scientific) and 10–20 μg proteins were used for immunoblot analysis. Cells cultured in vitro were lysed in the lysis buffer (10 mM Tris–Cl pH 8.0, 150 mM NaCl, 1 mM EDTA, 1% Triton X-100, 0.1% SDS, 0.1% Sodium deoxycholate) supplemented with protease inhibitor (Roche) and Phosphatase inhibitor cocktail (Pierce, Thermo Scientific). After centrifugation at max, 10 min at 4 °C, the cleared lysates were saved and protein concentrations were determined. Total protein of 10–20 μg were separated on NuPAGE Novex 4–12% Bis–Tris gel (Thermo Scientific) with MOPS running buffer (Thermo Scientific), and transferred to PVDF membrane (Bio-rad). After blocking with 5% non-fat skim milk (Difco, BD bioscience) in TBST (Tris-buffered saline with 0.05% Tween-20; Biosesang, Korea), the PVDF membrane were probed overnight with primary antibodies and then 1 h with HRP-conjugated secondary antibodies (Cell Signaling), followed by detection with SuperSignal West Pico or Femto Chemiluminescent substrate kit (Thermo Scientific). Protein extractions from paraffin-embedded sections were performed as described. 10–20 FFPE sections of 10 μm thickness were collected and de-paraffinized twice with Xylene, followed by rehydration and air-dry. After homogenizing in PEB buffer (100 mM Tris–Cl pH8.8, 2% SDS, 200 mM DTT) with motor pestles, sections were heated at 90 C for 2 h with constant shaking, followed by centrifugation for 20 min at 16,000 rpm at 4 °C. From the cleared lysates, protein concentrations were determined by BCA assay and 20–40 g protein were used for immunoblot analysis.

### Immunofluorescence imaging

Cells grown on glass cover slips were fixed in 4% PFA (paraformaldehyde) for 10 min, permeabilized and blocked with PBSA-0.3 T (BSA and triton-X 100 in PBS) for 30 min, and then incubated overnight with primary antibodies. After incubating with secondary antibodies conjugated with Alexa-488 or Alexa-594, nucleus was stained with DAPI. Prolong gold solution were used as the mounting media. LSM 700 confocal microscope (Zeiss) was used to observe the slides, acquire the images with the Zen software (Zeiss). Paraffin-embedded liver sections were processed, as like in IHC staining, and then detected with Alexa fluor-conjugated secondary antibodies. After nuclear staining with DAPI, the slides were mounted and the Confocal Laser Scanning Microscope LSM 700 and/or 780 (Zeiss) were used for acquisition and analysis of images.

### G-LISA assays

Activities of RhoA, Rac1, and cdc42 GTPase were measured by using G-LISA activation assay (Cytoskeleton) according to the manufacturer’s instructions. Serum-starved cells were stimulated with TGF-β1 for the indicated times. Cells were harvested and lysed on ice. After measuring protein concentration with Pierce BCA protein assay (Pierce, Thermo Scientific), the cell lysates corresponding to 500 μg/ml were subjected to 96 well of G-LISA assay, where the amounts of the bound active small GTPases were quantitated with primary antibodies, HRP-conjugated secondary antibodies, and color development. After normalization with that of 0 min, or with the untreated samples, the relative activation levels were presented.

### Luciferase reporter assays

To measure the activity of YAP/TAZ signaling, cells in 24-well culture plates were co-transfected with either 8xGIIC reporter (YAP reporter plasmid), or IFN-luc as negative control, and p*TK-Renilla* (Promega). After serum-starved and stimulated, luciferase activity was measured with a dual-luciferase assay kit (Promega) according to the manufacturer’s instructions. Renilla activity was used for normalization. For reporter assay in knockdown experiment, cells were transfected with siRNAs a day before transfecting with reporter plasmids.

### Reagents and antibodies

All three FAK family inhibitors (PF-431396, PF-562271, and PF-573228) and Pan-Src kinase inhibitor (Saracatinib) were purchased from Cayman Chemicals. Src kinase inhibitor PP2 and Smad3 inhibitor (SIS3) were purchased from Tocris of R&D Systems. ROCK inhibitor (Y-27632), Cdc42 GTPase inhibitor (ML-141), and Rac1/3 inhibitor (EHop-016) were purchased from Selleckchem. YAP-TEAD inhibitor (Verteporfin) was purchased from Cayman Chemicals (USA) and Selleckchem. Anti-FAK was purchased from Millipore. Antibodies against p-FAK(Y397), p-PYK2(Y402), PYK2, c-Src, p-Src(Y416), Smad2, p-Smad2(S465/467), p-Smad3(S423/425), Smad3, and YAP/TAZ were purchased from Cell Signaling Technology. anti-α-SMA and anti-actin were Sigma-Korea. Antibodies to detect p-PYK2(Y402), p-PYK2(Y579/580), and p-PYK2(Y881) were purchased from Invitrogen. Anti-CTGF and anti-myc-tag were purchased from Abcam. Anti-YAP and anti-α-SMA were purchased from Santa Cruz Biotechnology and Dako, respectively. Secondary antibodies to detect the primary antibodies were purchased as followed; HRP-conjugated anti-rabbit and anti-mouse from Pierce-Thermo Scientific, all Alexa-fluorophore conjugated antibodies from Invitrogen.

### Plasmids

Dominant negative forms of Rho family GTPase were purchased from Addgene as gifts of Dr. Gary Bokoch (Scripps Institute): pRK5-myc-RhoA-T19N (RhoA-DN, #12963), pRK5-myc-Rac1-T17N (Rac1-DN, #12984), and pRK5-myc-Cdc42-T17N (Cdc42-DN, #129730). IFN-luc was kindly provided by Dr. Wang-shik Ryu (Yonsei University, Korea). 8XGTIIC-luciferase (containing YAP/TAZ-responsive synthetic promoter; Addgene #34615) was a gift from Dr. Stefano Piccolo (University of Padova, Italy). pCMV-flag-YAP2-5SA (Constitutive active form of YAP; Addgene #27371) was a gift from Dr. Kunliang Guan (University of California San Diego, CA).

### Statistics

GraphPad Prism 5 (GraphPad Software) was used for all data analysis in this study. Statistical analyses between 2 groups were carried out by Student’s *t* test, with *p* < 0.05 considered as significant. Error bars were presented as standard deviation (SD).

## Results

### Inhibition of FAK/PYK2 suppresses liver fibrosis in vivo

**Figure 1:**
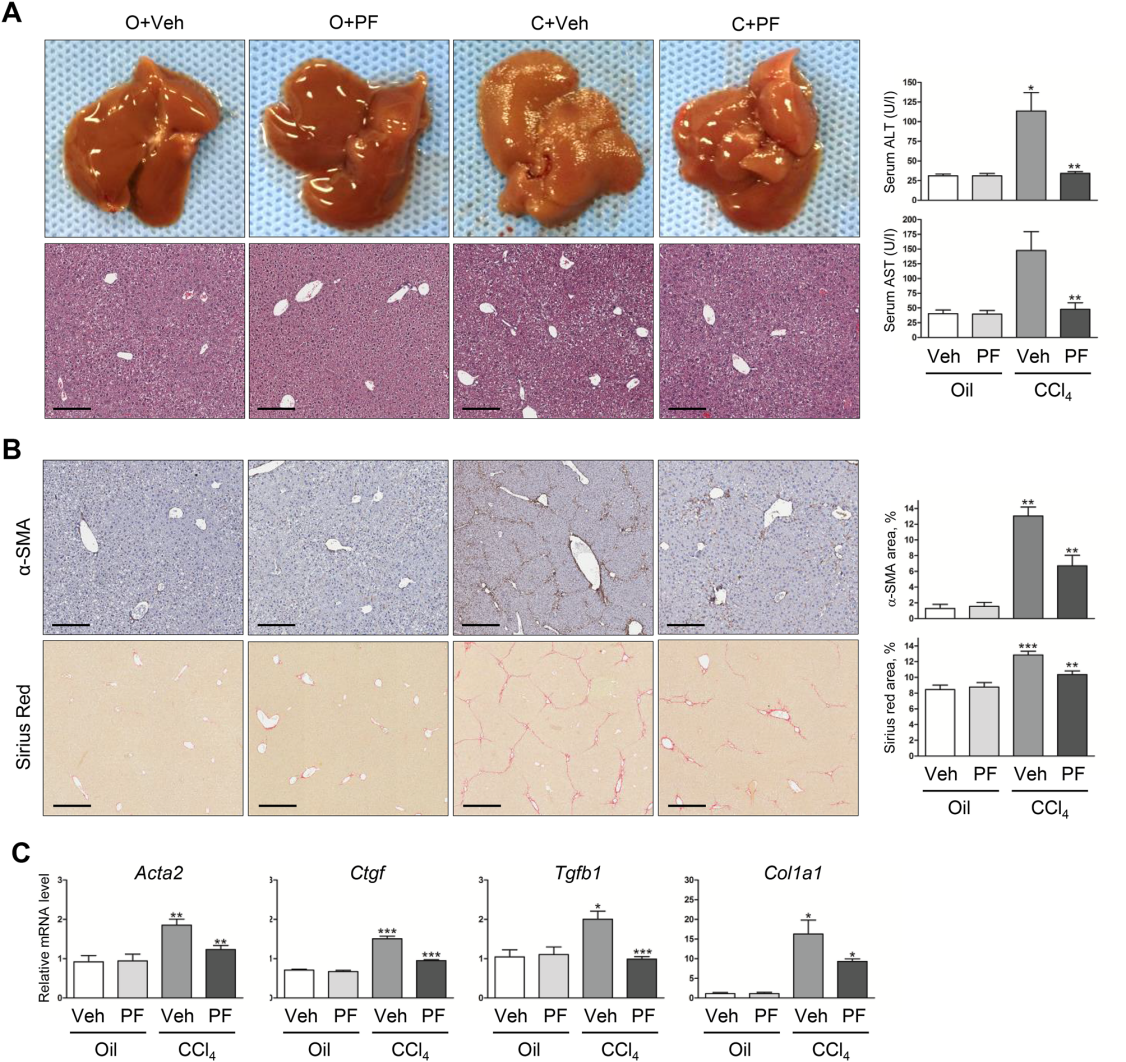
Treatment of the FAK/PYK2 inhibitor suppresses the hepatic fibrosis in CCl_4_-treated mice. Four groups of mice were treated; O + Veh (n = 4), O + PF (n = 5), C + Veh (n = 9), and C + PF (n = 9). (**A**) The whole livers and H&E stains of liver sections (n = 4–9) from each group. Scale bar: 200 μm. Serum ALT and AST levels in each groups shown as mean ± SD (n = 4–9). (**B**) IHC of α-SMA (for activated HSCs; n = 4) and Sirius red (for fibrillary collagen; n = 4–9). The stained areas were shown as percentages of the total area of each liver sections (n = 4–9 in each groups). Scale bar: 200 μm in the upper row and 400 μm in the lower row. (**C**) qPCR of Acta2, Ctgf, Tgfb1, and Col1a1 mRNA shown as fold change compared with O + Veh control (n = in each groups). Student's t-test of C + Veh to O + Veh and C + PF to C + Veh was performed; *p < 0.05, **p < 0.01, and ***p < 0.001. O, Oil; Veh, Vehicle; C, CCl_4_; PF, PF-431396.

To address whether HSCs interact with ECM through focal adhesion to promote liver fibrosis, we used a CCl_4_-induced liver fibrosis mice model. Administration of CCl_4_ in 6 weeks resulted in liver fibrosis, while co-administration of CCl_4_ and PF-431396, a dual pharmacological inhibitor of FAK and PYK2, significantly suppressed such liver injuries (Fig. 1A). The reduction of liver injuries by PF-431396 was also evidenced by the lowered serum levels of alanine aminotransferase (ALT) and aspartate aminotransferase (AST) (Fig. 1A). Immunohistochemical staining of the paraffin-embedded sections and its quantitation showed that α-SMA accumulation, a marker of activated HSC, on CCl_4_ administration was significantly suppressed in the PF-431396-treated group (Fig. 1B, top row). Furthermore, fibrillar collagen staining with Sirius-red revealed marked development of periportal fibrosis with numerous fibrous septa spreading into the lobules and perivascular space as well as portal-portal bridges of septa (Fig. 1B, bottom row). However, the characteric findings of liver fibrosis was mitigated in PF-431396-treated mice (Fig. 1B), suggesting collagen accumulation induced by CCl_4_ was suppressed by inhibition of FAK/PYK2. Furthermore, quantitative RT-PCR results showed that upregulation of several pro-fibrotic genes (*Acta2*, *Col1a1*, *Tgfb1*, and *Ctgf*) in CCl_4_-treated groups was also suppressed by co-administration of PF-431396 (Fig. 1C). Taken together, these results indicate pharmacological inhibition of FAK family kinase suppresses CCl_4_-induced liver fibrosis in mice.

### PYK2, but not FAK, plays a central role in TGF-β1-mediated induction of CTGF

**Figure 2:**
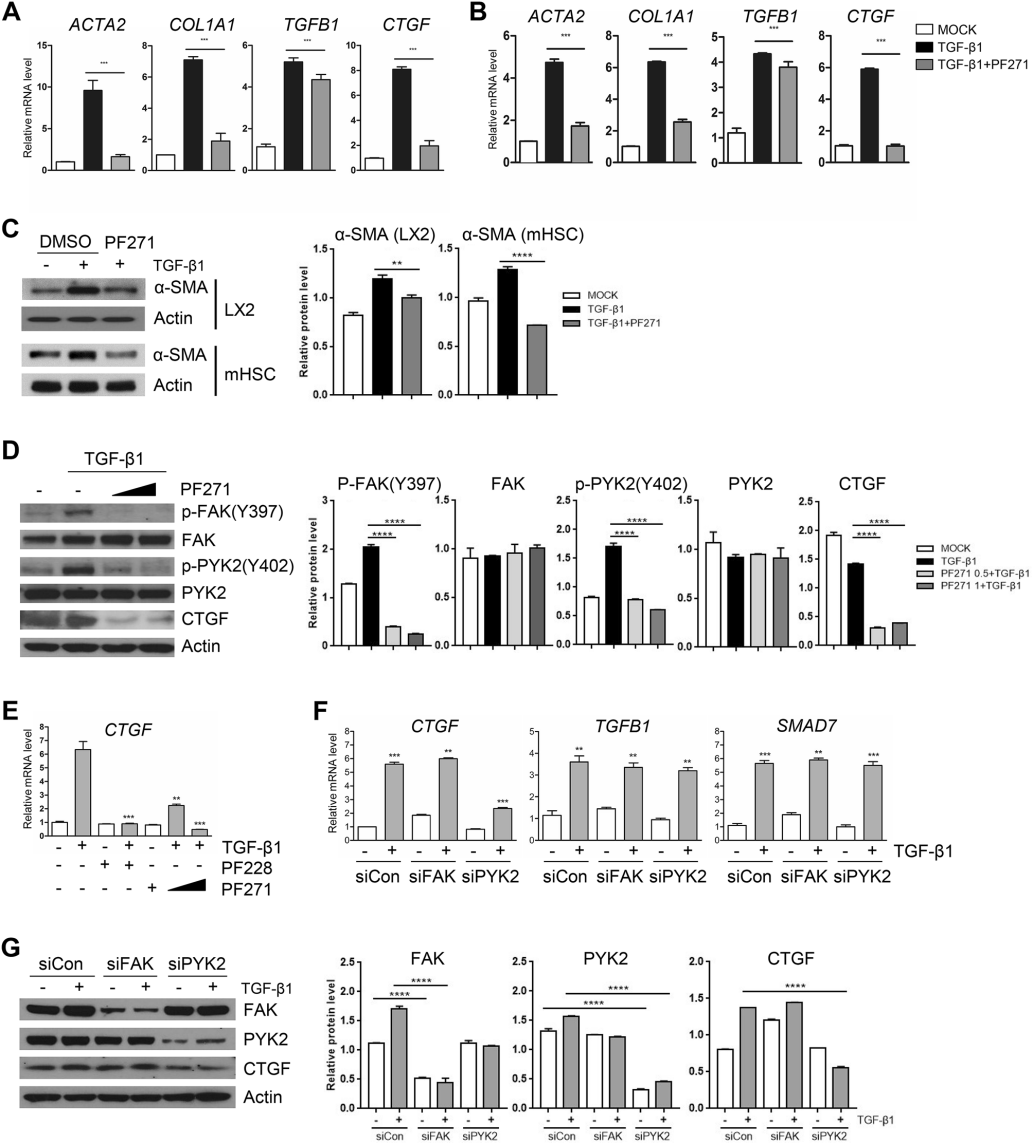
Inhibition of PYK2, but not FAK, suppresses upregulation of pro-fibrotic CTGF expression on stimuli of TGF-β1. (**A**, **B**) qPCR of fibrogenic gene expression in in vitro activated primary mouse hepatic stellate cell (mHSC) (**A**) and in LX2 (**B**), treated as indicated and shown as fold change (n = 3). (**C**) Western blot of α-SMA in mHSC and LX2 after treatments. (**D**) Western blot of activated forms of both FAK and PYK2, and CTGF in LX2 treated as indicated (n = 3). (**E**) qPCR of CTGF expression in LX2 that were treated with either of two FAK/PYK2 inhibitors (n = 3). (**F**, **G**) After transfection with siRNAs of negative control, FAK or PYK2, qPCR of the indicated gene expression (**F**) and western blot (**G**) were performed. Two FAK/PYK2 dual inhibitors were used: PF271, PF-573271; PF228, PF573228. Student's t-test; *p < 0.05, **p < 0.01, and ***p < 0.001.

To investigate the mechanism underlying suppression of CCl_4_-induced liver fibrosis by FAK family kinase inhibitor, we tested its effects in vitro using mouse primary HSCs (mHSCs) that were isolated and in vitro-activated, as well as in LX-2 cells, an immortalized human HSC cell line. As evidenced by qPCR, short treatment (1–2 h) of TGF-β1 increased the expression of pro-fibrogenic genes in both mHSC and LX-2, whereas pre-treatment with PF-573271, another dual inhibitor of FAK/PYK2, suppressed TGF-β1-mediated induction of the pro-fibrogenic genes (Fig. 2A,B), consistent with the in vivo results (Fig. 1C). Interestingly, we also observed that the upregulation of TGFB1 mRNA was not significantly suppressed by the inhibitor, implying that FAK family kinases might play differential roles in pathways downstream of TGF-β1 stimuli. Furthermore, upregulation of α-SMA protein by TGF-β1 in both mHSCs and LX-2 was also suppressed by PF-573271 (Fig. 2C). Western blotting analysis also demonstrated that the PF-573271 inhibited phosphorylation of both FAK at residue Y397 and PYK2 at residue Y402 in the kinase domain, and confirmed that the increased level of CTGF protein in response to TGF-β1 stimuli was diminished by PF-573271 (Fig. 2D). We found that *CTGF* expression was upregulated as early as less than 1 h after TGF-β1 stimuli, which was similar to the induction of *TGFB1* and *SMAD7* (data not shown). In order to rule out the possibility of off-target effect of PF-271, we also tested PF-228, another FAK/PYK2 inhibitor. qPCR results demonstrated that TGF-β1-mediated upregulation of CTGF was greatly suppressed by the pre-treatment of two different FAK family inhibitors, PF-271 and PF-228, with similar efficiencies (Fig. 2E). Since FAK and PYK2 share about 40–60% similarity in amino acid sequence and similar domain organization, their functional redundancy has been widely accepted. In contrast, evidences are growing to support the ideas of differential functions in several cancer types and cancer stem cells. To determine whether the two FAK family members might play distinct roles in *CTGF* induction on fibrogenic TGF-β1 stimuli, we employed siRNA-mediated knock-down; siRNA against *FAK* (siFAK) or *PYK2* (siPYK2) achieved more than 80%, or 90%, respectively (Fig. 2G). While knockdown of *PYK2*, but not of *FAK*, dramatically suppressed TGF-β1-induced *CTGF* expression in comparison with siControl-transfected cells, both *TGFB1* and *SMAD7* mRNA expression were unaffected by the both knockdowns (Fig. 2F). Western blot also confirmed that TGF-β1-mediated upregulation of *CTGF* protein level was specifically inhibited by siRNA of *PYK2*, but not of *FAK* (Fig. 2G). These data implicate that activation of PYK2, but not FAK, is essential for the pathway for *CTGF* expression. In addition, the signaling pathway downstream of TGF-β1 for *CTGF* expression is distinct from that for *TGFB1* and *SMAD7* expression.

### PYK2-mediated CTGF induction by TGF-β1 is Smad-independent

**Figure 3:**
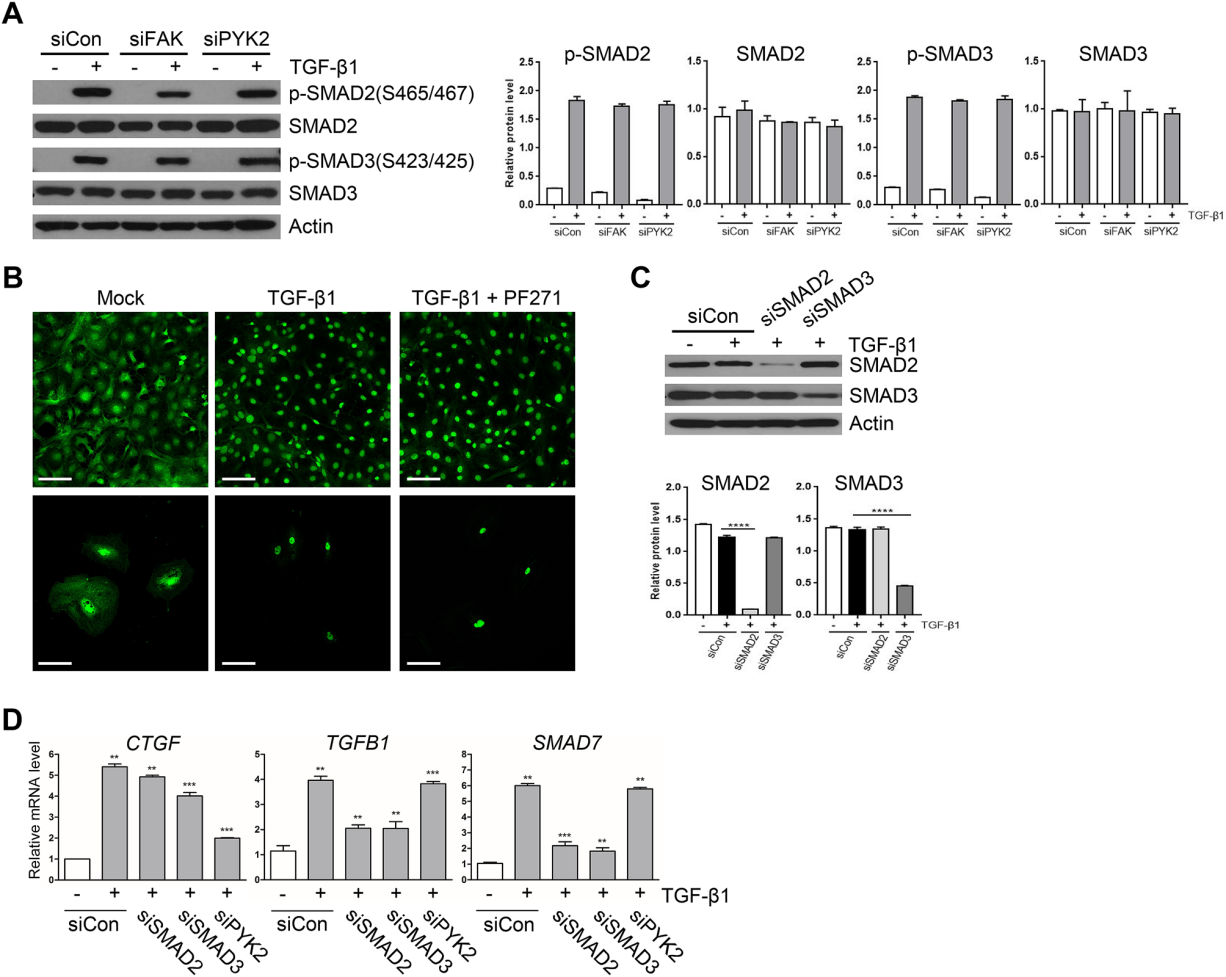
PYK2 mediated CTGF induction by TGF-β1 is independent of Smad activation. (**A**) Western blot of activated forms of Smad2 and Smad3 upon TGF-β1 stimuli in LX2 transfected with siRNAs of negative control, FAK, or PYK2 (n = 3). (**B**) IF of Smad2 in LX2 grown on glass and treated as indicated (n = 4). Scale bar: 50 μm. (**C**) Western blot of Smad2 and Smad3 (n = 2) and (**D**) qPCR of indicated genes (n = 3) in LX2 transfected with siRNAs of control, Smad2, Smad3, or Pyk2. Student's t-test; *p < 0.05, **p < 0.01, and ***p < 0.001.

It is generally known that TGF-β1 signaling pathways are either Smad-dependent or -independent, leading to different target gene expression; one for expression of *TGFB1*, *SMAD7*, and *NOX4* is Smad-dependent, while one for *CTGF* expression is both dependent and independent on Smad activation. To investigate whether PYK2 plays roles in TGF-β1 signaling pathways in Smad-dependent or -independent manner, we either knockdowned or inhibited PYK2 and measured the Smad activation by TGF-β1. Western blot showed that knockdown of PYK2 with siRNA did not change the levels of phosphorylation of both Smad2 and Smad3 upon TGF-β1 stimuli (Fig. 3A). In addition, Immunofluorescence staining of Smad proteins showed that the amount of cytosolic Smad proteins were significantly reduced upon TGF-β1 treatment, and pretreatment with PF-271 did not change the translocation of Smad proteins at both LX2 and activated mHSC (Fig. 3B). Furthermore, siRNA-mediated knockdowns of Smad2 and Smad3 significantly down-regulated the expression of both *TGFB1* and *SMAD7*, whereas *CTGF* induction was weakly affected by the knockdowns, but not as much as siPYK2 treatment (Fig. 3D). Western blot showed that siRNAs efficiently reduced Smad2 and Smad3 proteins in 80% and 60%, respectively (Fig. 3C). SIS3, a selective inhibitor of Smad3 phosphorylation, also significantly reduced expression levels of TGFB1 and SMAD7, but only slightly affected CTGF levels (data not shown). These data suggested that PYK2 plays roles in Smad-independent TGF-β1 signaling pathways.

### Association of PYK2 phosphorylation with liver fibrosis in human and mouse

**Figure 4:**
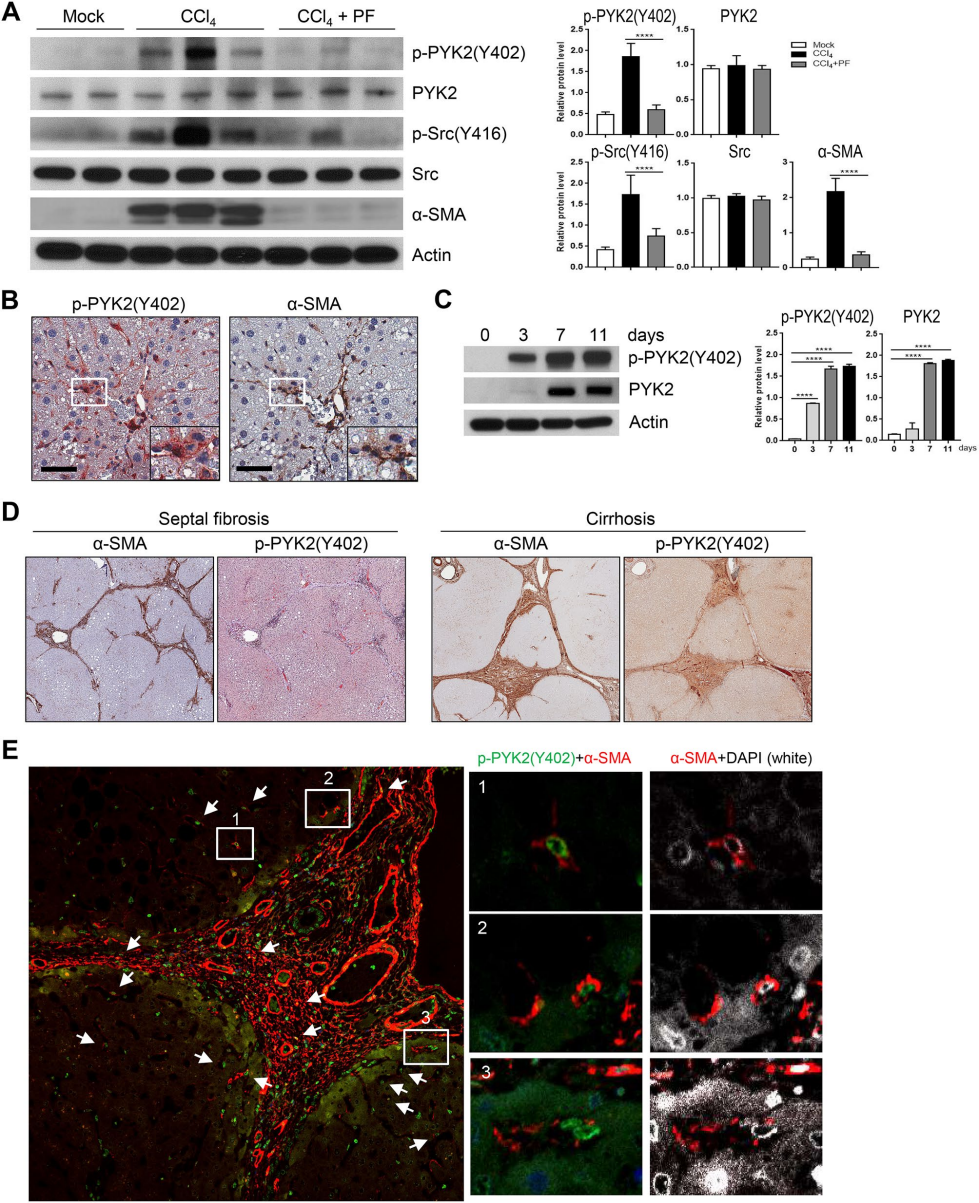
PYK2 activation is associated with liver fibrosis of human and mouse. (**A**) Western blot of activated forms of PYK2 and SRC, and α-SMA expression from livers of O + V (n = 2), CCl_4_ (n = 3), and CCl_4_ + PF (n = **3**). (B) IHC of paraffin-embedded liver sections from CCl_4_-treated mice; p-PYK2(Y402)-AEC staining, followed by α-SMA-DAB with hemotoxylin couterstain (n = 4). (**C**) Western blot of both the activated and the total of PYK2 in primary mouse HSC that were cultured in vitro at the indicated times. (**D**) IHC of p-PYK2(Y402) and α-SMA in paraffin liver sections from patients diagnosed with septic fibrosis (n = 3) or cirrhosis (n = 3). (**E**) ICC/IF of cirrhosis patient liver sections (n = 3) stained for p-PYK2(Y402) (green), α-SMA (red), and DAPI (white). Arrows and insets represent high-magnification images of cells having nuclear stains of p-PYK2(Y402) with cytoplasmic α-SMA. Magnification, ×400 in (**B**) and in SF section of (**D**), and ×200 in cirrhosis section of (**D**). Scale bar: 500 μm in (**B**), 500/1000 μm in (**D**), and 100 μm in (**E**).

To further confirm the significance of the above in vitro findings of primary mHSC and LX2, we measured PYK2 activation in CCl_4_-treated mouse livers, in comparison with inhibitor or mock-treated mouse livers. Western blots evidenced that the level of p-PYK2(Y402), an activated autophosphorylated form, was increased in CCl_4_-treated group, as well as α-SMA expression, whereas PF-431396 significantly suppressed their induction (Fig. 4A). It is noteworthy to say that phosphorylation on lysine 416 of SRC, the activated form, was also significantly increased in CCl_4_-treated group and reduced by PF-431396 treatment (Fig. 4A). SRC kinase has been known to recognize the activated form of PYK2 and further phosphorylate on lysine 579, 580, and 881, thereby facilitating downstream signaling pathway of PYK2. Moreover, IHC of mouse CCl_4_-treated liver sections displayed that p-PYK2(Y402) strongly localized to the region having high α-SMA expression, mainly at both portal tracts and sinusoids (Fig. 4B), indicating that the activated HSCs have increased levels of p-PYK2(Y402). We also found that p-PYK2(Y402) localized both nucleus and cytoplasm at sinusoidal region, in consistent with the previous report showing nuclear localization of PYK2 with unknown biological significance. During in vitro activation of isolated primary mHSC, p-PYK2(Y402) level significantly increased in time-dependent manner with the peak of 7–11 days, as well as total PYK2 (Fig. 4C). To further confirm the significant role of PYK2 activation in hepatic fibrogenesis, we stained paraffin-embedded patient liver sections with diagnosis of septal fibrosis (n = 7) and cirrhosis (n = 7). IHC and IF evidenced that in accordance with the mouse fibrotic liver staining, p-PYK2(Y402) were significantly concentrated to fibrotic septa and sinusoids having high α-SMA expression, with minor stains around parenchyma (Fig. 4D,E). Here again, the p-PYK2(Y402)-positive nucleus was surrounded by α-SMA-positive cytoplasm both in septa area and in sinusoids. Together, these data suggested that the activation of PYK2 is clearly evident at HSC activated to express α-SMA.

### Src acts downstream of PYK2 for TGF-β1-mediated CTGF induction

**Figure 5:**
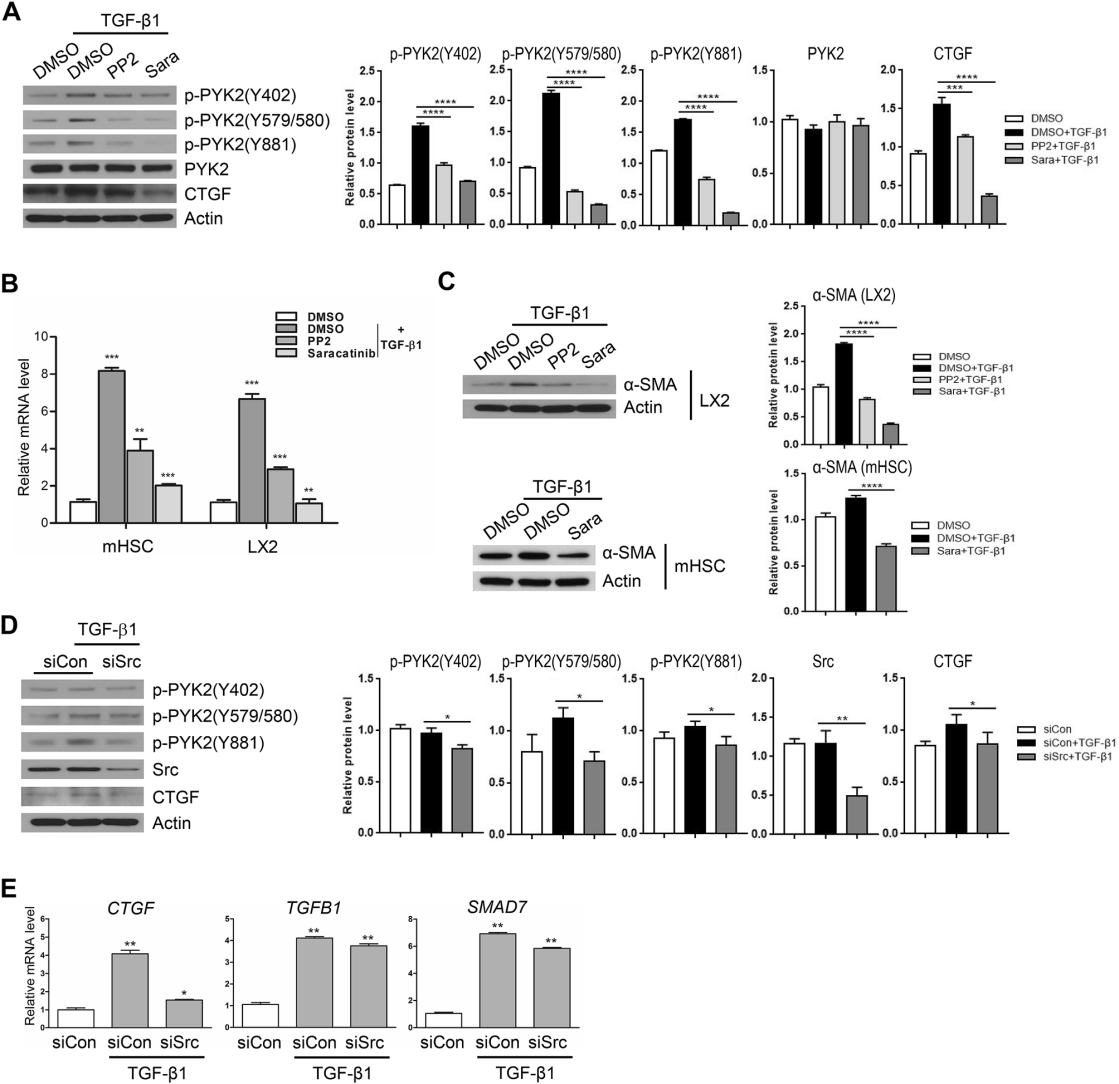
PYK2-Src activation is required in TGF-β1 mediated CTGF upregulation. (**A**) Western blot of different phosphorylated forms of PYK2 as well as CTGF in LX2 pretreated with two SRC inhibitors (PP2 and Saracatinib) (n = 3). (**B**) qPCR of CTGF mRNA expression (shown as fold change, n = 3) and (**C**) Western blot of α-SMA in LX2 treated as indicated (n = 2). (**D**) Western blot of CTGF and different phosphorylated forms of PYK2 (n = 2) and (**E**) qPCR of CTGF, TGFB1, and SMAD7 mRNA expression (shown as fold change, n = 3) in LX2 transfected with siRNA of control or Src. Student's t-test; *p < 0.05, **p < 0.01, and ***p < 0.001.

At the initial activation, PYK2 auto-phosphorylates its lysine 402 residue, and then the p-Y402 residue is recognized by Src kinase that further phosphorylate other tyrosine residues (579, 580, 881 residues) of PYK2. Previously we showed that Src activation (detected by phosphorylation on lysine 416) was significantly increased in CCl_4_ treatment, but was comparable to mock treatment in PF-431396 co-treated groups (Fig. 4A). Therefore it is conceivable that Src might play downstream of PYK2 in TGF-β1-mediated CTGF induction. Western blot showed that pretreatment with two SRC family kinase inhibitors (PP2 and Saracatinib) followed by TGF-β1 abolished phosphorylation of tyrosine residues 579/580/881 down below the level of no treatment, whereas phosphorylation of tyrosine residue 402 was only slightly decreased (Fig. 5A). Concomitantly, CTGF induction was also shown to be decreased by 60% at PP2 and by 80% at Saracatinib, as seen by western bot and by qPCR at both LX2 and mHSC (Fig. 5A,B). Accordingly, increased expression of α-SMA on TGF-β1 stimuli was suppressed by the Src inhibitors at both LX2 and mHSC (Fig. 5C). To further confirm the involvement of Src kinase in TGF-β1-mediated CTGF induction, we employed siRNA-mediated knockdown of Src protein, and found that siRNA-treated cells showed 60–70% reduction in Src proteins (Fig. 5D). As expected, the knockdown decreased not only phosphorylation on tyrosine residues of PYK2, but also suppressed TGF-β1-mediated upregulation of CTGF (Fig. 5D). Interestingly, the knockdown resulted in more than 85% reduction in *CTGF* mRNA, but less than 10% in *TGFB1* mRNA and 20% in *SMAD7* mRNA, as determined by qPCR (Fig. 5E). We also found that CTGF induction by ectopic expression of constitutively active Src mutant having Y527F (Src-CA) was also suppressed by knock-down of PYK2 (Supplementary Figure S1). Thus, these data conclusively demonstrate that PYK2 activation and subsequent recruitment and signal amplification via feed-forward mechanism among PYK2-Src-RhoA triad is essential for TGF-β1-mediated induction of CTGF.

### Activation of PYK2-Src-RhoA triad is essential for CTGF induction

**Figure 6:**
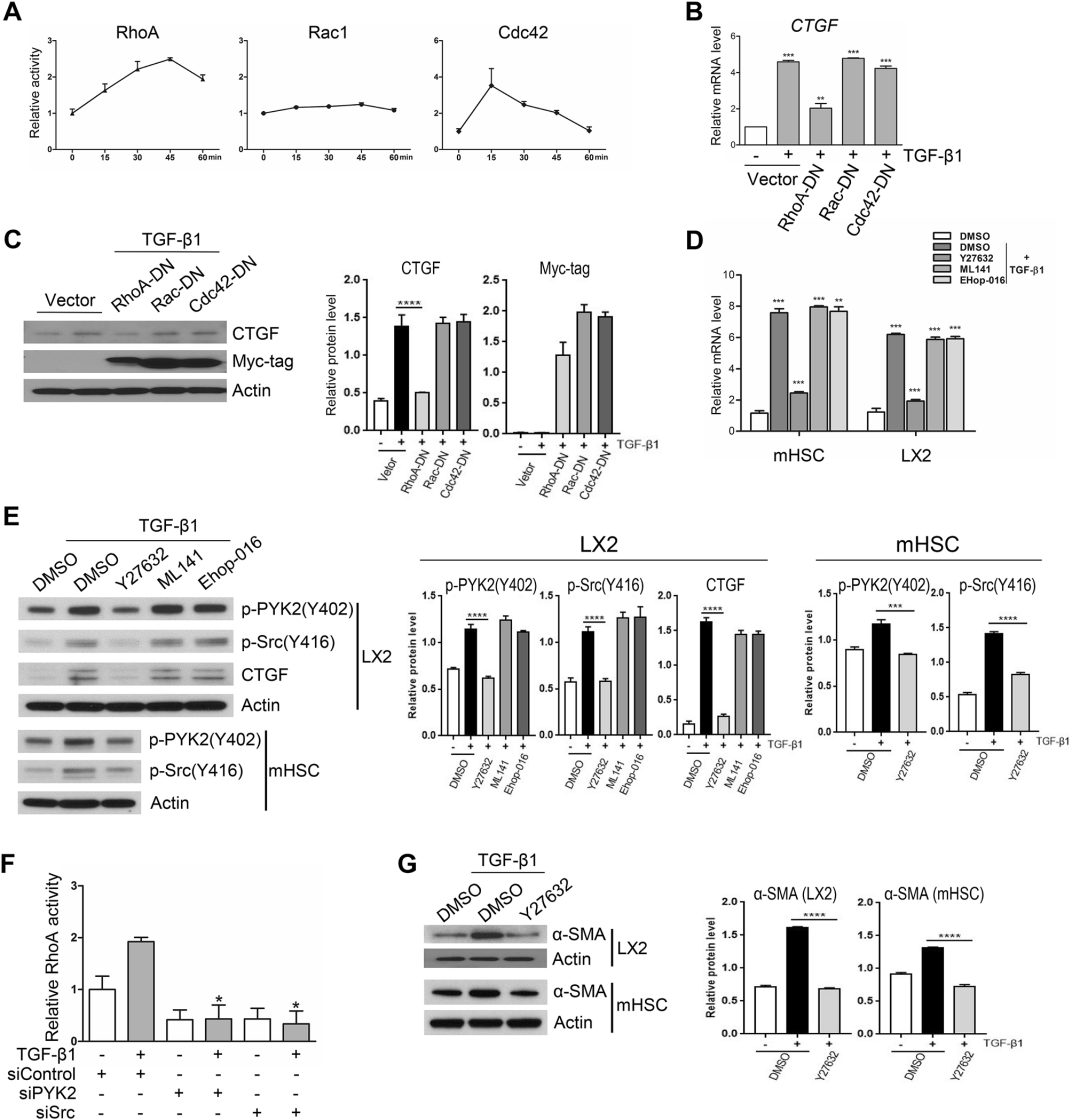
Activation of PYK2-Src-RhoA triad is essential for TGF-β1 mediated upregulation of CTGF. (**A**) G-LISA activation assay of Rho Small GTPase family in LX2 at different time points after TGF-β1 stimuli and shown as fold change (n = 4). (**B**) qPCR of CTGF mRNA expression (shown as fold change, n = 3) and (**C**) western of CTGF (n = 2) in LX2 transfected to overexpress the dominant-negative forms of Rho GTPase family members and followed by TGF-β1 stimuli. (**D**) qPCR of CTGF mRNA expression (shown as fold change, n = 3) and (**E**) western of activated forms of both PYK2 and Src, and CTGF (n = 2) in both activated mouse hepatic stellate cells and LX2 pretreated with Rho family inhibitors. (**F**) G-LISA assay of RhoA activation by TGF-β1 stimuli for 30 min in LX2 transfected with siRNA of control, PYK2, or Src (n = 3). (**G**) Western of α-SMA in both LX2 and activated primary mouse hepatic stellate cells that were pretreated with ROCK inhibitor Y27632 before TGF-β1 stimuli (n = 2). Student's t-test; *p < 0.05, **p < 0.01, and ***p < 0.001.

It has been demonstrated that activation of RhoA GTPase by TGF-β1 is one of several Smad-independent pathways, and RhoA-ROCK activity is required for the dynamics of focal adhesion (FA) and for Src recruitment to FA during cell adhesion and migration. To determine whether Rho family GTPase activities are involved in CTGF induction by TGF-β1, we first measured activities of RhoA, Rac, and Cdc42 using G-LISA assay in time-course. While RhoA activities quickly increase after addition of TGF-β1 with a peak of 45 min, Rac1 activity showed no response, and Cdc42 showed its peak activity at 15 min and then decreased back to the basal level at 60 min (Fig. 6A). Next, we ectopically overexpressed dominant-negative (DN) forms of RhoA, Rac, and Cdc42, and then measured CTGF induction by TGF-β1, and found that only RhoA-DN significantly suppressed CTGF induction, as determined by qPCR and western blot (Fig. 6B,C). To further confirm the exclusive role of RhoA in CTGF induction, we pre-treated both LX2 and mHSC with inhibitors before TGF-β1 stimuli (Y27632, Rho-associated protein kinase (ROCK) inhibitor; ML-141, Cdc42 inhibitor; Ehop-016, Rac1/3 inhibitor). In consistent with the ectopic expression results, qPCR and western blot showed significant suppression of CTGF induction only at Y27632-treated cells (Fig. 6D,E). Interestingly, we also found that the phosphorylated forms of PYK2 at Y402 and of Src at Y416 were also decreased down to the basal level in Y27632-treated LX2, as well as mHSC cells (Fig. 6E), suggesting that RhoA activation is mutually involved in PYK2-Src signaling. To address the possibility that both PYK2 and Src is required for RhoA activation upon TGF-β1 stimulation, we measured RhoA activities after siRNA-mediated knockdown of PYK2 or Src. In comparison with siRNA of control, both siPYK2 and siSrc blunted RhoA activation upon TGF-β1 stimuli, as well as suppressed the basal activation levels (Fig. 6F), indicating that PYK2-Src activation is essential for TGF-β1-mediatd RhoA activation. To address the possibility that RhoA activation in turn promotes PYK2-Src signaling in a feed-forward mechanism, we assessed CTGF induction in PYK2-knockdown cells upon treatment with the RhoA activator calpeptin. Treatment with the RhoA activator alone efficiently increased CTGF induction that was blocked by PYK2-knockdown (Supplementary Figure S2). Moreover, it also increased the levels of the phosphorylated forms of both PYK2 and Src even in the absence of TGF-β1 stimuli (Supplementary Figure S3). These data suggest that not only PYK2-Src activation is essential for TGF-β1-mediated RhoA activation, but also that RhoA-ROCK activation in turn promotes PYK2-Src activation in a feed-forward mechanism, that might result in signal amplification. Of note, Y27632 abolished α-SMA induction of TGF-β1 in both LX2 and mHSC cells (Fig. 6G). Together, these data demonstrate that TGF-β1 stimuli results in activation of PYK2-Src-RhoA triad for CTGF induction.

### TGF-β1-induced CTGF expression is dependent on PYK2/Src/RhoA/YAP activation

**Figure 7:**
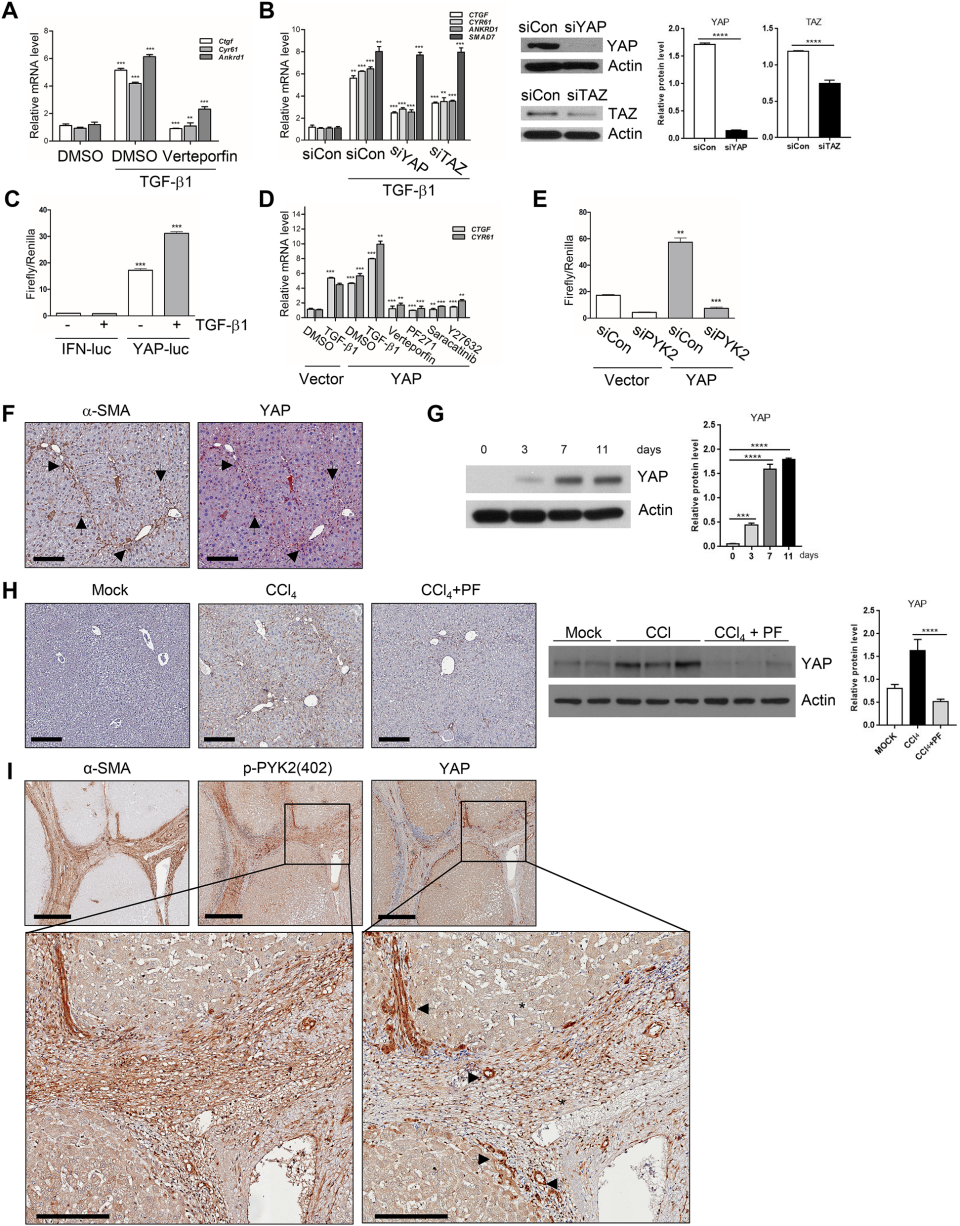
Profibrogenic TGF-β1 activates YAP in PYK2-dependent manner in vivo and in vitro. (**A**) qPCR of YAP target genes expression in activated mHSC that were pretreated with DMSO or Verteporfin before TGF-β1 stimuli, and shown as fold change (n = 3). (**B**) qPCR of YAP target genes and SMAD7 mRNA expression (shown as fold change, n = 3) and western blot of YAP and TAZ (n = 2) in LX2 transfected with siRNA of control, YAP, or TAZ, followed by TGF-β1 stimuli. (**C**) YAP reporter assay in LX2 transfected with either IFN-luc or YAP-luc (8xGTIIC-luc) followed by TGF-β1 stimuli as indicated (n = 3). (**D**) qPCR of CTGF and CYR61 mRNA expression in LX2 that were transfected with either vector alone or YAP(wt) and then followed by the treatments as indicated (n = 3). (**E**) YAP reporter assay in LX2 transfected with siRNA of either control or PYK2, followed by 2nd transfection with vector alone or YAP(wt) (n = 2). (**F**) IHC co-staining of α-SMA and YAP in paraffin liver sections of CCl_4_-treated mouse (n = 4). Arrows indicate the co-localized regions. (**G**) Western of YAP expression in primary mHSCs at the indicated times on in vitro culture. (**H**) IHC of YAP in CCl_4_-treated mouse livers (n = 3) and western blot of YAP expression in livers from O + V (n = 3), O + CCl_4_ (n = 3), CCl_4_ + PF (n = 3). (**I**) IHC of α-SMA, p-PYK2(402), and YAP expression in serial liver sections from patients with cirrhosis (n = 4). The enlarged insets were shown below. The luciferase activities were normalized with co-transfected renilla luciferase activity. Scale bar: 200 μm in (**F**, **H**), 500 μm in (**I**), and 200 μm in insets of (**I**), Student's t-test; *p < 0.05, **p < 0.01, and ***p < 0.001. Magnification, ×100 in (F, H) and ×40 in (**I**).

CTGF is the hepatic pro-fibrogenic modulator in several organs including liver and was upregulated immediately in response to TGF-β1 (Fig. 2) in activated HSCs. In addition, the above results demonstrated that activation of PYK2-SRC-RhoA triad is essential for CTGF upregulation on TGF-β1 stimuli in hepatic fibrogenesis. In addition, *CTGF* is also one of representative target genes of the transactivator protein YAP/TAZ, and YAP/TAZ activation has been reported to play important roles in fibrosis of several organs. Therefore, it is conceivable that TGF-β1 activates the transcriptional regulators YAP/TAZ through PYK2-SRC-RhoA triad for *CTGF* induction in activated HSCs. To demonstrate the possibility whether TGF-β1 treatment activates YAP/TAZ, we first measured the expression of the target genes in mHSCs that were stimulated with TGF-β1 in the absence or presence of Verteporfin, specific inhibitor of YAP/TAZ-mediated transcription activity. As shown by qPCR results, it was evidenced that TGF-β1 induced *CTGF*, *Cyr61,* and *Ankrd1* in mHSC, and the induction was significantly suppressed by Verteporfin treatment (Fig. 7A). Knock down of YAP or TAZ by siRNA in LX2 also suppressed TGF-β1-mediated YAP/TAZ target gene induction with no effect on upregulation of *SMAD7* (Fig. 7B), indicating that profibrogenic *CTGF* induction by TGF-β1 is dependent on YAP/TAZ. Moreover, the reporter assay using YAP-luc (8XGTIIC-luc) further evidenced that addition of TGF-β1 upregulated activities of YAP/TAZ by twofold in comparison with control treatment (Fig. 7C). The high reporter activities by YAP-luc at the mock treatment might be due to the intrinsic activation of YAP/TAZ in activated HSCs, as consistent with IF results showing that YAP protein predominantly localized to the nucleus at both LX2 and mHSC (data not shown). To further demonstrate that PYK2-SRC-RhoA triad plays roles in signaling pathway driven by YAP/TAZ activation, we measured target gene expression on ectopic expression of YAP protein followed by treatments of each inhibitors (Fig. 7D). As expected, overexpression of YAP alone induced both *CTGF* and *Cyr61* mRNA expression as similar degree to TGF-β1 treatment alone, and combination of TGF-β1 and YAP overexpression increased the target gene expression much more than either TGF-β1 alone or YAP overexpression alone did. However, treatment of Verteporfin, PF-271, Saracatinib, or Y27632 all significantly abolished YAP overexpression-mediated *CTGF* and *Cyr61* induction (Fig. 7D), suggesting that PYK2-Src-RhoA triad is essential for YAP-mediated target gene expression. Moreover, siRNA-mediated knock down of PYK2 abolished the reporter activities driven by ectopically expressed YAP (Fig. 7E). In consistent with these in vitro data, we also found that YAP protein levels were increased during in vitro activation of isolated primary mHSC in time-dependent manner (Fig. 7F). The chronic mouse liver injuries induced by CCl_4_ treatments resulted in increased YAP proteins in comparison with mock-treated group, while co-treatment of PF-431396 nullified it, as demonstrated by western blot (Fig. 7G). Accordingly, YAP stain in paraffin liver sections significantly increased in CCl_4_-treated groups, whereas co-treatment of PF-431396 greatly attenuated the increase (Fig. 7H). Interestingly, YAP stains in CCl_4_-treated liver sections highly localized to the portal triad and fibrotic area where α-SMA are co-localized, indicating that YAP protein levels are increased at in vivo activated HSCs (Fig. 7H). In addition, IHC of serial liver sections of patients diagnosed with cirrhosis (n = 4) showed that α-SMA-positive stroma area were also strongly positive for both pPYK2(Y402) and YAP (Fig. 7I), indicating that activated human HSCs also expressed high levels of phosphorylated PYK2 and YAP as well as α-SMA.

**Figure 8:**
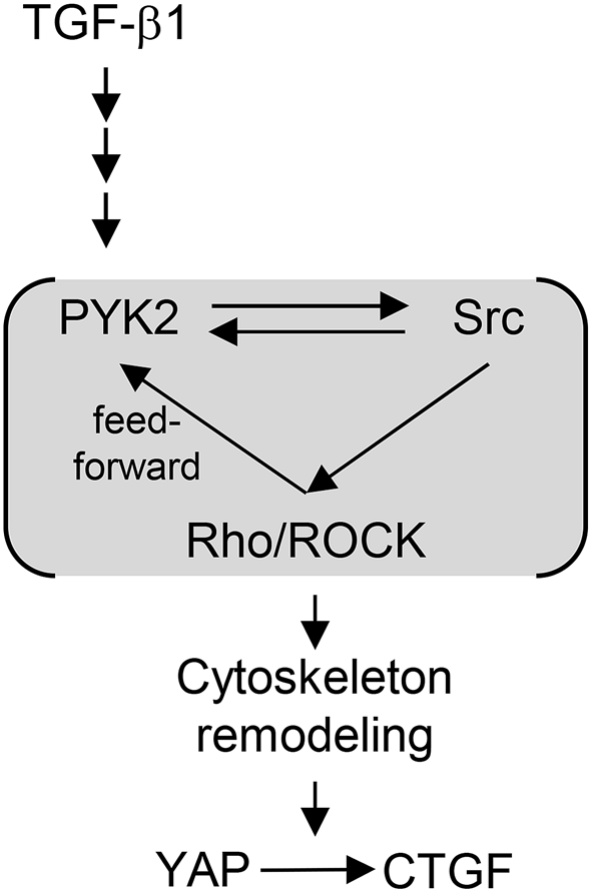
Diagram of PYK2-Src-RhoA triad functioning between TGF-β1 and YAP activation. TGF-β1 activates PYK2, and autophosphorylated PYK2 recruits and activates Src, which results in activation of RhoA. Upon RhoA activation, PYK2 and Src is further activated in a feedforward manner leading to signal amplification.

Collectively, these data demonstrate that TGF-β1-induced activation of PYK2-Src-RhoA triad leads to YAP/TAZ activation for *CTGF* induction during hepatic fibrogenesis (Fig. 8).

## Discussion

Liver fibrosis is characterized by dysregulated production of ECM by activated HSCs^1,2^. The interaction between HSCs and ECM through focal adhesion mediated by FAK family kinases is one of the important steps during the process of liver fibrogenesis^24,27^. To date, the role of PYK2 during the process of liver fibrosis has been questioned with inconsistent reports. In the present study, we investigated the role of PYK2 in liver fibrogenesis by pharmacological inhibition of FAK family kinases in vitro and in vivo, and confirmed activation of PYK2 in human liver specimens from patients diagnosed with septal fibrosis or cirrhosis. The significant finding of this study is that activation of PYK2, but not FAK, is essential for TGF-β1-mediated induction of CTGF during HSC activation.

To extend our studies on the role of PYK2 in hepatic fibrogenesis to an in vivo context, activation of PYK2 was assessed in a mouse model of CCl_4_-induced liver fibrosis^28,29^, which recapitulates several features of human liver fibrosis including septal fibrosis or cirrhosis. Indeed, our animal study revealed the presence of phosphorylated residue Y402 of PYK2 supporting the critical role of PYK2 activation in liver fibrogenesis (Fig. 4). We further confirmed our findings in human liver specimens from patients diagnosed with septal fibrosis or cirrhosis.

CTGF is a key pro-fibrogenic mediator of TGF-β1-mediated ECM production^9–11^. However, the signal transduction mechanism by which CTGF exerts has been unclear. In the present study, we report that PYK2-mediated CTGF expression by TGF-β1 signaling is distinct from the canonical Smad-dependent TGF-β1 signaling pathway (Fig. 3), and is dependent on PYK2-Src-RhoA-YAP activation (Fig. 5).

It has been demonstrated that activation of RhoA GTPase by TGF-β1 is one of several Smad-independent pathways^30^, and RhoA-ROCK activity is required for the dynamics of focal adhesion and for Src recruitment to FA during cell adhesion and migration^31^. We assessed whether Rho family GTPase activities are involved in CTGF induction by TGF-β1, and from our experiments we have demonstrated that TGF-β1 stimuli results in activation of PYK2-SRC-RhoA triad for CTGF induction (Fig. 6).

Because the interaction between HSCs and ECM through focal adhesion is one of the key steps for TGF-β1-mediated CTGF induction, the FAK family kinases represent potential targets for anti-fibrosis therapy. In particular, the PYK2-dependence of TGF-β1-mediated CTGF induction has therapeutic implications for liver fibrosis.

TGF-β induces HSC to express and deposit ECM components and also secret more TGF-β1 for an autocrine signal^32,33^. Among the several stimuli for YAP activation, major upstream regulator of YAP/TAZ activity is the mechanical stress, including stiffness of ECM^34,35^. YAP mechano-senses and mediates signaling from extracellular ECM back to HSC to regulate several biological processes, such as proliferation, adhesion, and migration^36^. Therefore, it is conceivable that TGF-β signaling and YAP signaling might concomitantly occur and crosstalk within HSC. Both human and mouse HSC showed that TGF-β1 upregulates expression of YAP target genes (CTGF, CYR61, and ANKRD1) and reporter activity (Fig. 7).

TGF-β1 signaling has two branches; Smad-dependent pathway, or Smad-independent^12^. It has been reported that Smad2/3 makes complexes with YAP and translocate to nucleus^4^. However, inhibition of Smad2 and Smad3 using siRNA and pharmacological inhibitor had no effect on upregulation of CTGF on TGF-β1 treatment at both human and mouse HSC (Fig. 2 and data not shown). EMT processes primarily initiate by breaking up cell–cell junctions where several junction molecules has been known to decrease YAP protein stability directly or activating the Hippo kinase cascade. However, EMT induction by TGF-β is also Smad-dependent. There are several pathways induced by TGF-β1 Smad-independently, leading to activation of PI3K, p38MAPK, JNK, Erk, and Rho GTPases^30^. We showed that TGF-β1 treatment induced RhoA activity and CTGF induction was abolished by inhibitor of Rho-associated kinase and ectopic expression of dominant negative RhoA (Fig. 6), suggesting that TGF-β1 induces RhoA activation leading to actin remodeling and further YAP activation. However, the mechanism of RhoA activation by TGF-β1 has been in vague.

Cell-ECM interaction occurs via integrin complex through “inside-out” and/or “outside-in” signaling^17^. The cytoplasmic region of integrin b interact with several signal mediators, including integrin-linked kinase, FAK, PYK2, and also with actin binding proteins linking the integrin complex to cytoskeleton networks. ILK inactivates several components of hippo kinase cascade, leading to YAP activation^7^. FAK is also known to activate YAP^23^. The contribution of PYK2 to YAP signaling has not been reported. Our results showed that TGF-β1 treatment promoted both FAK and PYK2 activation (Fig. 2), but YAP activation by TGF-β1 was suppressed only by siRNA-mediated knock-down of PYK2, not of FAK, in target gene expression and reporter assay (Fig. 7). We also obtained the same results in stable cell lines of PYK2 knockdown (data not shown). PYK2 has long been believed to play redundant roles of FAK, but low similarity in amino acid sequences outside the kinase domain of PYK2 and FAK and subcellular localization implicated their distinct functions^19^. One major difference of PYK2 from FAK is that increase in calcium level promotes PYK2 activation. Indeed, transient treatment of A23187, calcium ionopore, dramatically increased CTGF expression in HSC without TGF-β, and only siRNA of PYK2 abolished the effect of A23187 (data not shown). In addition, our data showed that PYK2-Src-RhoA triad mediates the convergence of TGF-β1 and YAP (Figs. 4, 5, 6).

Regarding the mechanism of PYK2, not FAK, for the crosstalk, different sets of heterodimer of integrin a/b form might have distinct affinities or efficiencies to them, thereby presumably leading to different downstream signaling pathways. Based on different subcellular localization of PYK2 and FAK, it is also conceivable that they might form different cell adhesions, such as focal complex, focal adhesion, lamellipodia, filopodia, and invadopodia, or effect on the dynamics of the adhesions^37^. Since both PYK2 and FAK localize to nucleus, they might aid nuclear localization of YAP with different efficiencies.

This crosstalk of TGF-β and YAP signaling might work in a feed-forward manner; TGF-β1 induces ECM deposition that is sensed back to HSC by YAP to promote HSC proliferation and produce more TGF-β1 and ECM. At the early stages of hepatic fibrosis, less ECM deposition might end up with weak YAP activation, while more ECM deposition at the later stages of fibrosis might lead to strong YAP activation accelerating the rate of ECM deposition and exacerbate the disease progression. Terminating the feed-forward crosstalk of TGF-β1 and YAP signaling might be a novel way of therapeutic strategy of liver fibrosis and cirrhosis. Our results showed that inhibition of PYK2 was successfully suppressed both target gene expression and reporter activity of YAP on stimuli of TGF-β1 and overexpression of YAP (Fig. 7). Therefore, PYK2 may serve as a novel therapeutic target for liver diseases, including fibrosis cirrhosis, and even hepatocellular carcinoma.

In summary, we identified TGF-β1-induced activation of PYK2-Src-RhoA triad leads to YAP/TAZ activation for CTGF induction in liver fibrosis. Targeting PYK2 may be an attractive target for developing novel therapeutic strategies for the treatment of liver fibrosis.

## Supplementary information

Supplementary information.

## Abbreviations

HSC: Hepatic stellate cells
TGF: Transforming growth factor
CTGF: Connective tissue growth factor
FAK: Focal adhesion kinase
CCl_4_: Carbon tetrachloride
YAP: Yes-associated protein
ALT: Alanine aminotransferase
AST: Aspartate aminotransferase
α-SMA: Alpha-smooth muscle actin
ROCK: Rho-associated protein kinase
