# Supplementary material for: Proline-rich tyrosine kinase 2 mediates transforming growth factor-beta-induced hepatic stellate cell activation and liver fibrosis
Source: Sci Rep. 2020 Dec 3;10:21018. doi: 10.1038/s41598-020-78056-0 (PMC7713048; doi:10.1038/s41598-020-78056-0)

Supplementary Material

Title:

Proline-rich tyrosine kinase 2 mediates transforming growth factor-beta-induced hepatic stellate cell activation and liver fibrosis

Authors:

Jonghwa Kim,[1,*] Wonseok Kang,[1,*] So Hee Kang,[1] Su Hyun Park,[1,2] Ji Young Kim, [1,2] Sera Yang,[1] Sang Yun Ha,[3] Yong-Han Paik[1,2]

[1]Department of Medicine, Samsung Medical Center; Department of Health Sciences and Technology, SAIHST, Sungkyunkwan University School of Medicine, Seoul, Korea. [2]Department of Health Sciences and Technology, SAIHST, Sungkyunkwan University, Seoul, Korea. [3]Department of Pathology, Samsung Medical Center, Sungkyunkwan University School of Medicine, Seoul, Korea.

* These authors contributed equally.

Supplementary Material

Supplementary Table S1. Target gene-specific Taqman primers for qPCR.

Gene	Species	Cat #
Acta2	mouse	Mm01546133_m1
Col1a1	mouse	Mm00801666_g1
Tgfb1	mouse	Mm03024053_m1
Ctgf	mouse	Mm01192933_g1
Cyr61	mouse	Mm00487498_m1
Ankrd1	mouse	Mm00496512_m1
Gapdh	mouse	Mm99999915_g1
ACTA1	human	Hs00559403_m1
COL1A1	human	Hs00164004_m1
TGFB1	human	Hs00998133_m1
CTGF	human	Hs01026927_g1
SMAD7	human	Hs00998193_m1
CYR61	human	Hs00155479_m1
ANKRD1	human	Hs00173317_m1
18S	human	Hs99999901_s1

Supplementary Material

Supplementary Table S2. siRNA sequences.

Gene	Species	Sequence (5’ – 3’)
FAK	human	GGACAGCGUGAGAGAGAAA
PYK2	human	CCAUGGAGCAAGAGAGGAA
SRC	human	AAGCAACUUGCCCAGCUAUGA
SMAD2	human	GUCCCAUGAAAAGACUUAA
SMAD3	human	1) GGAGAAAUGGUGCGAGAAG<br>2) CUCUGACGCUUGUGACAGU
YAP	human	AAGAAGUAUCUCUGACCAG
TAZ	human	AGGUACUUCCUCAAUCACA

Supplementary Material

Supplementary Figure S1. Knock-down of PYK2 suppresses CTGF expression induced by ectopic expression of constitutively active Src protein. LX2 cell lines was transfected with either siRNA of control or PYK2, and then followed by 2nd round transfection with either Vector (pcDNA 3.1) or Src-CA (Y527F, a gift from David Shalloway (Addgene, #17675)). After 48 hr post transfection, CTGF expression was monitored by RT-qPCR (n=2). Student's t-test; *p<0.05, **p<0.01, and ***p<0.001.

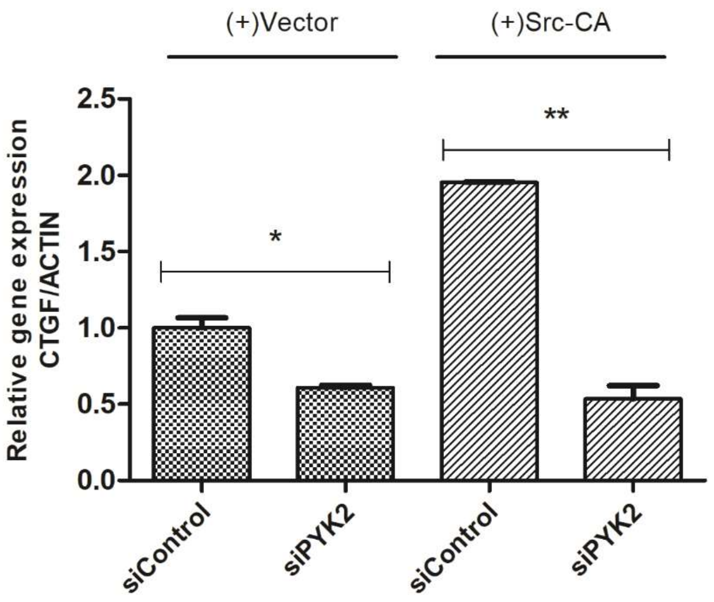

Supplementary Material

Supplementary Figure S2. Knock-down of PYK2 suppresses CTGF induction on treatment with known Rho activator. LX2 cells were transfected with either siRNA of control or PYK2, and then followed by 2hr treatment of Rho activator (50uM of Calpeptin, a major component of CN-01), along with TGF-β1 (5ug/ml). CTGF expression was monitored by RT-qPCR (n=2). Student's t-test; *$p<0.05$, **$p<0.01$, and ***$p<0.001$.

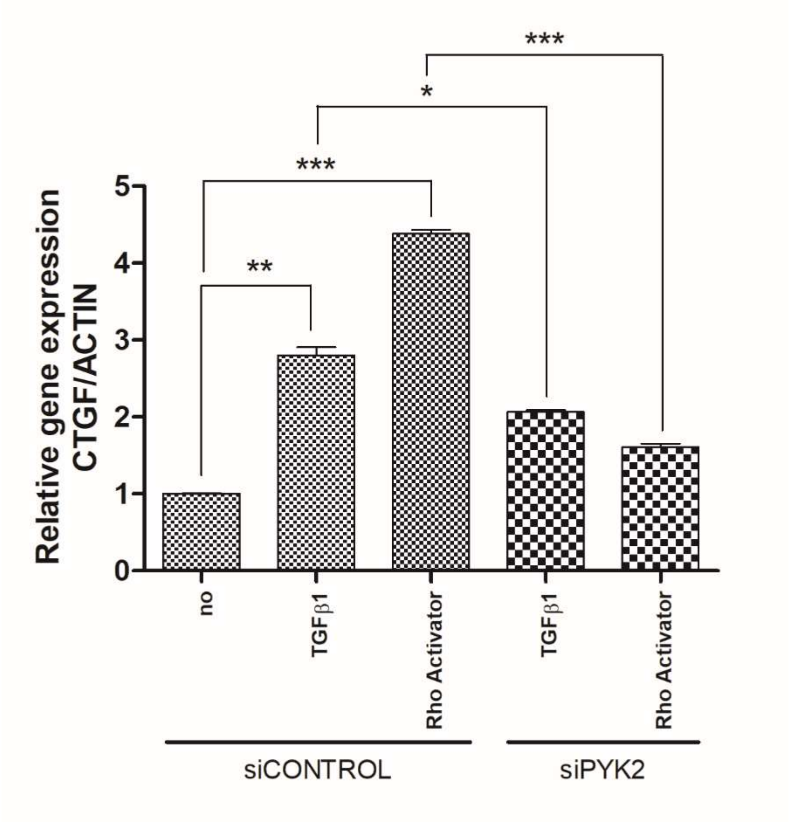

Supplementary Material

Supplementary Figure S3. Rho activator treatment alone was sufficient to increase Pyk2 auto-phosphorylation as well as Src activation. LX2 was treated with 10uM or 50uM of Rho activator (Calpeptin) for 15, 30, 45, or 60 min. Western blot was performed to monitor the phosphorylated forms of Pyk2 and Src.

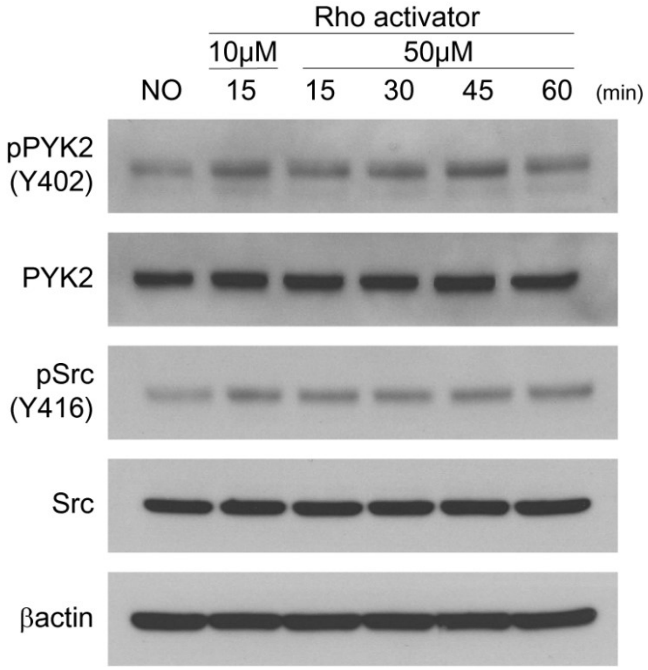

Supplementary Material

Gel scans from the main figures

Figure 2. C

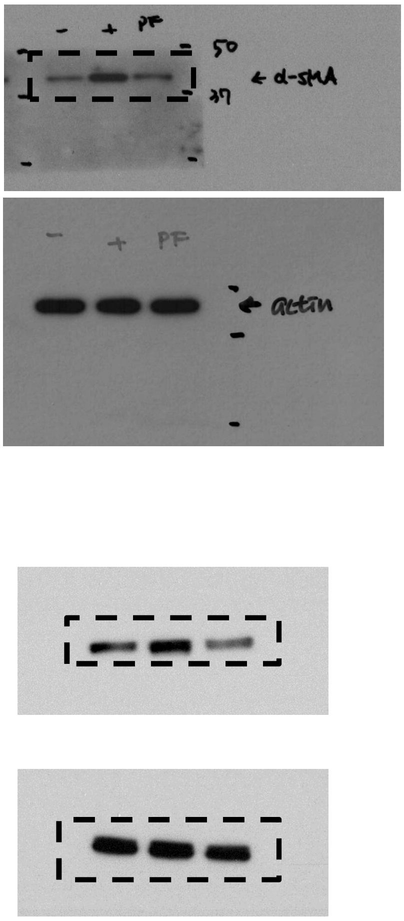

Figure 2. D

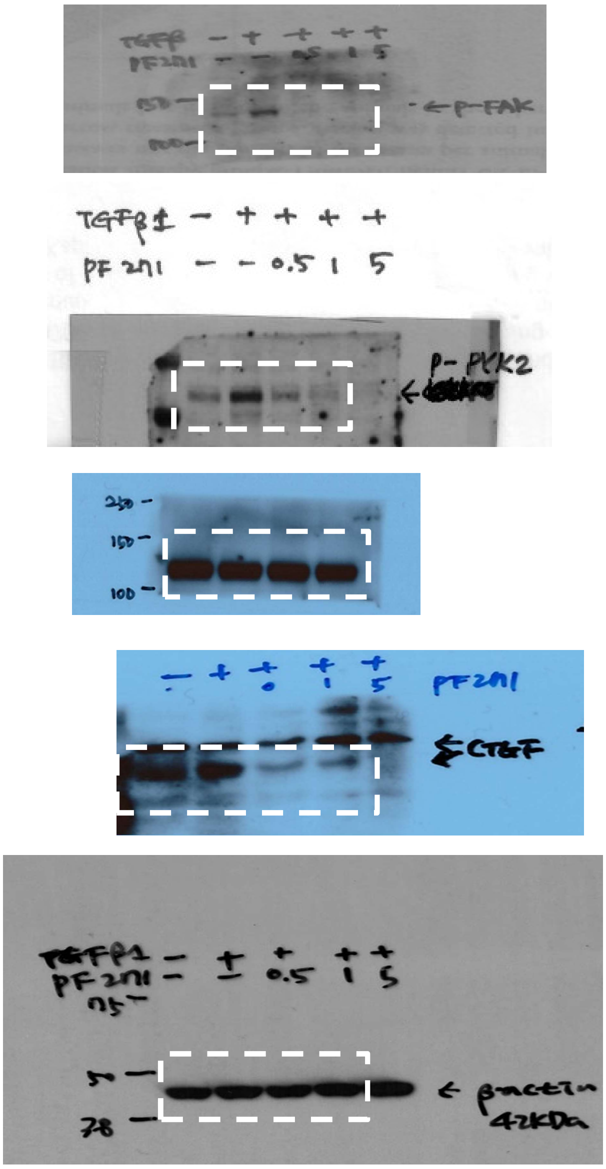

Supplementary Material

Gel scans from the main figures

Figure 2. G

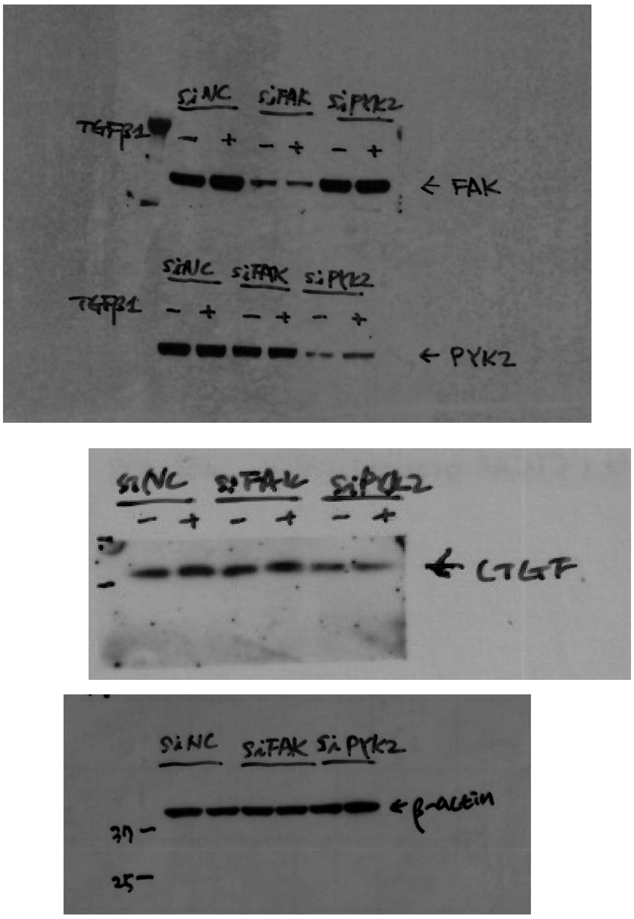

Supplementary Material

Gel scans from the main figures

Figure 3. A

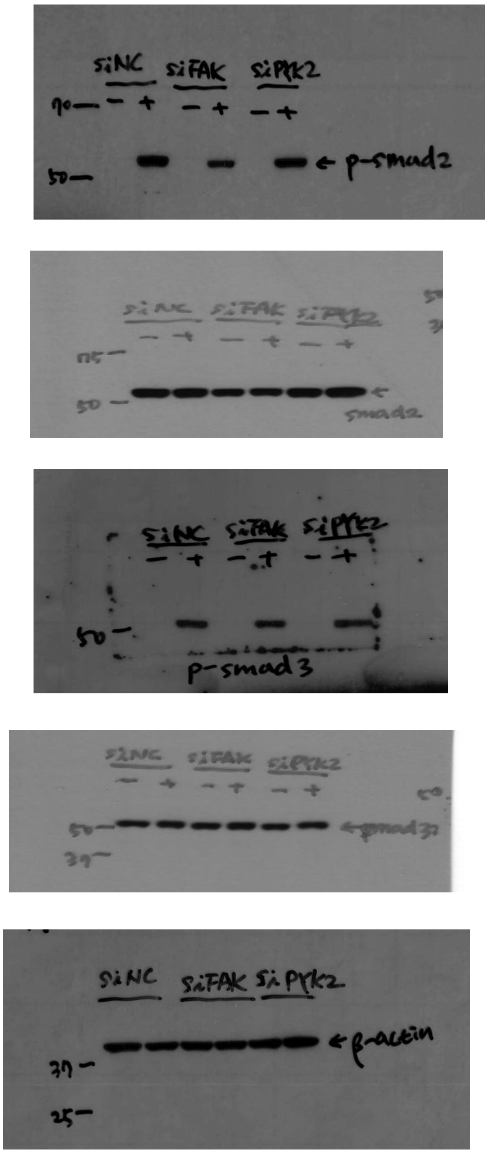

Figure 3. C

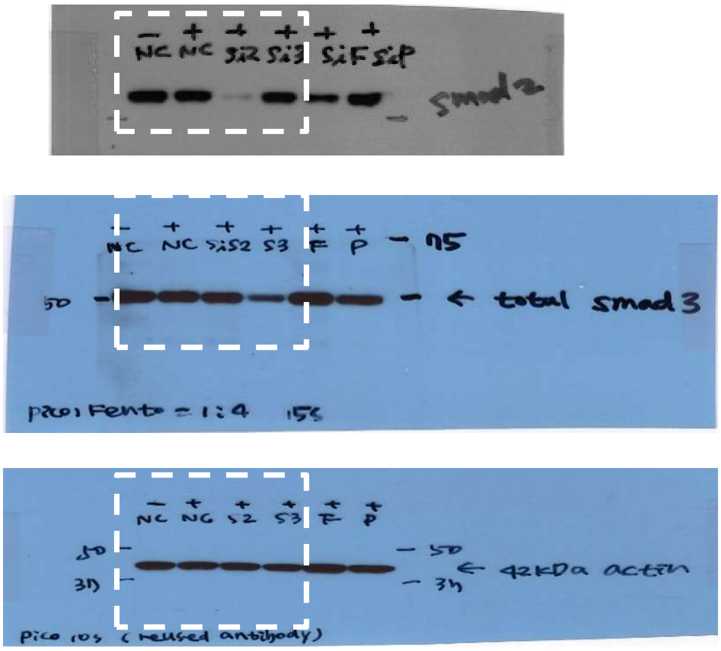

Supplementary Material

Gel scans from the main figures

Figure 4. A

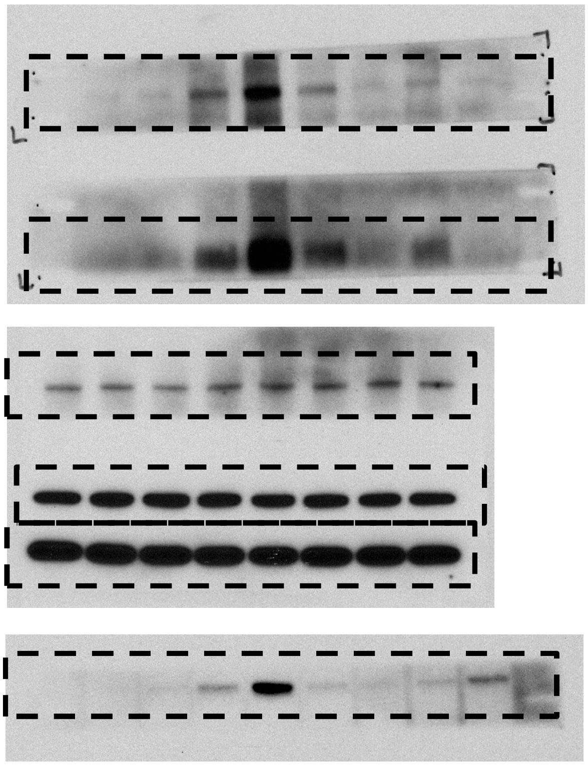

Figure 4. C

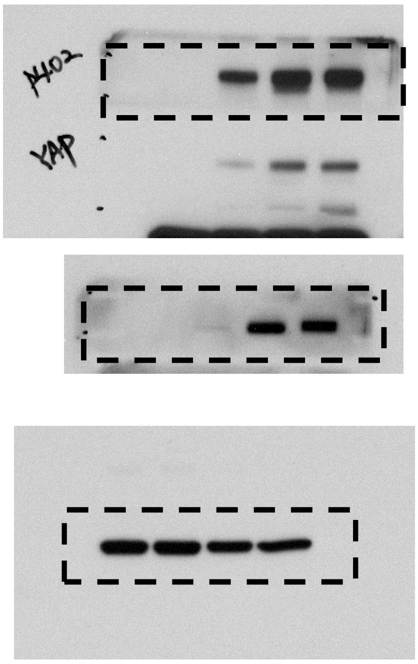

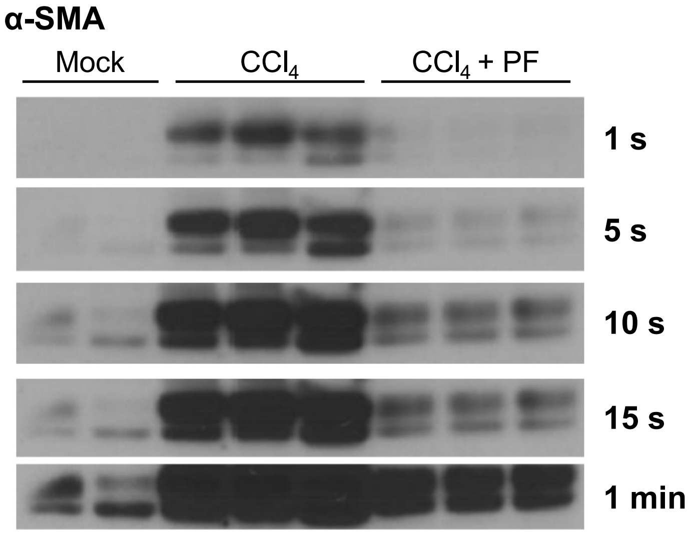

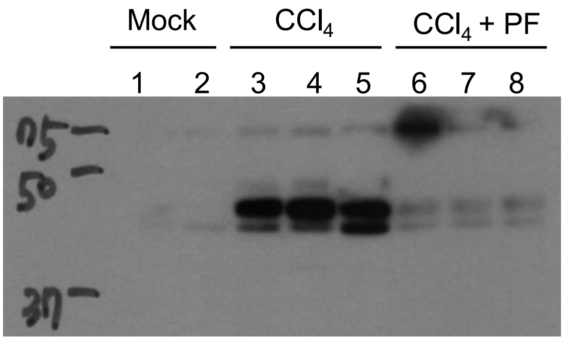

Supplementary Material

Gel scans from the main figures

Figure 5. A

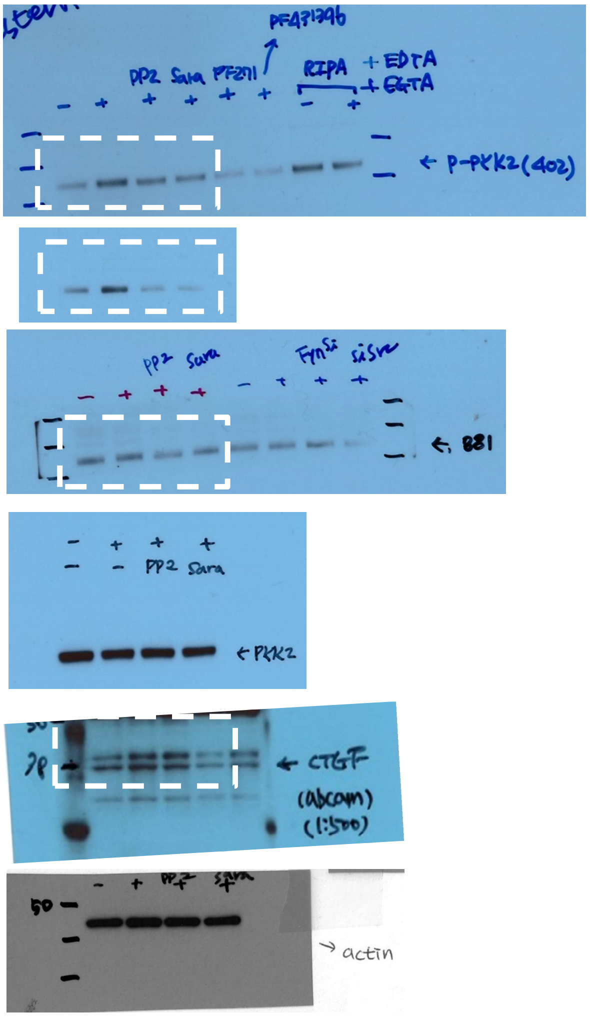

Figure 5. C

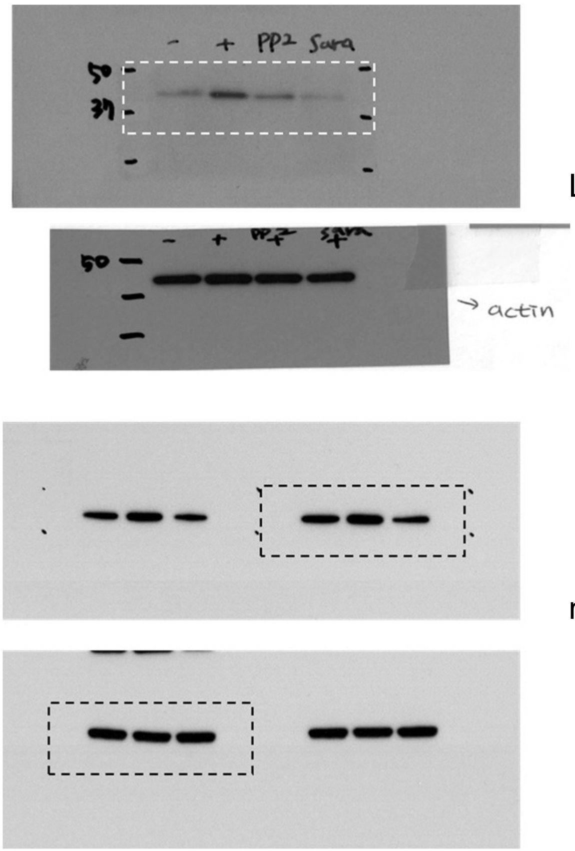

LX2

mHSC

Supplementary Material

Gel scans from the main figures

Figure 5. D

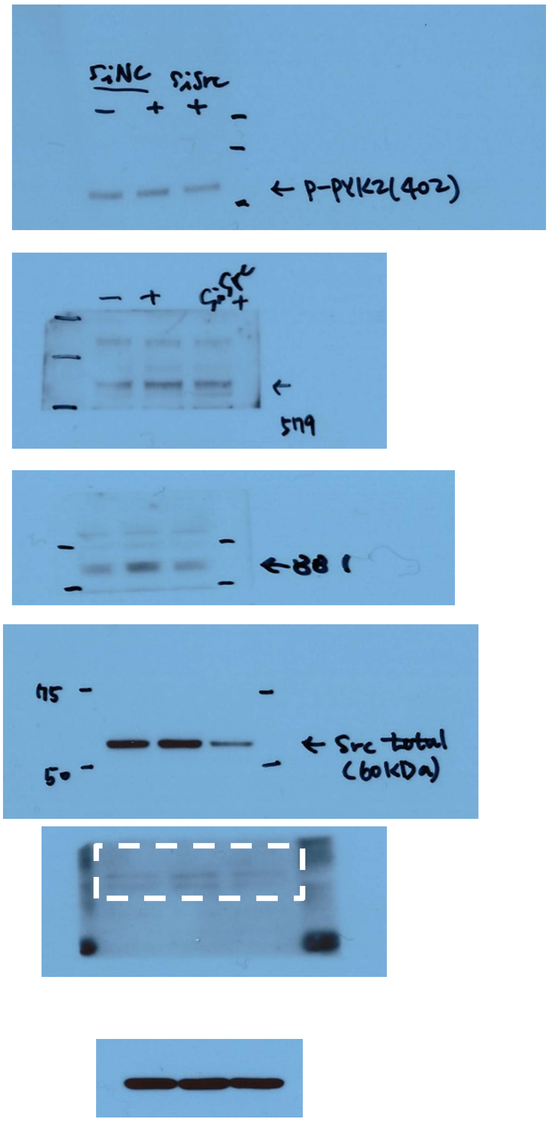

Supplementary Material

Gel scans from the main figures

Figure 6. C

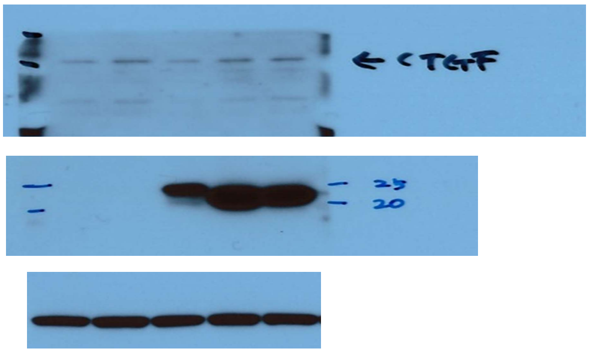

CTGF

Myc-tag

Actin

Figure 6. E

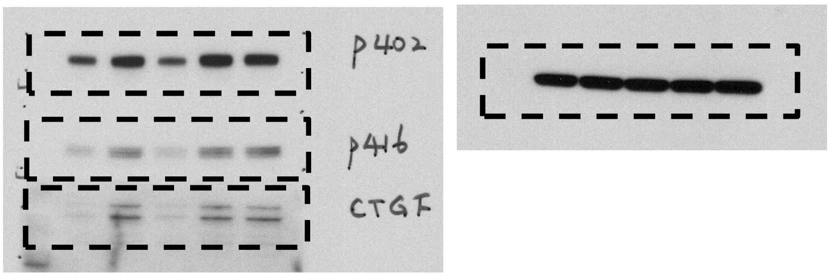

LX2

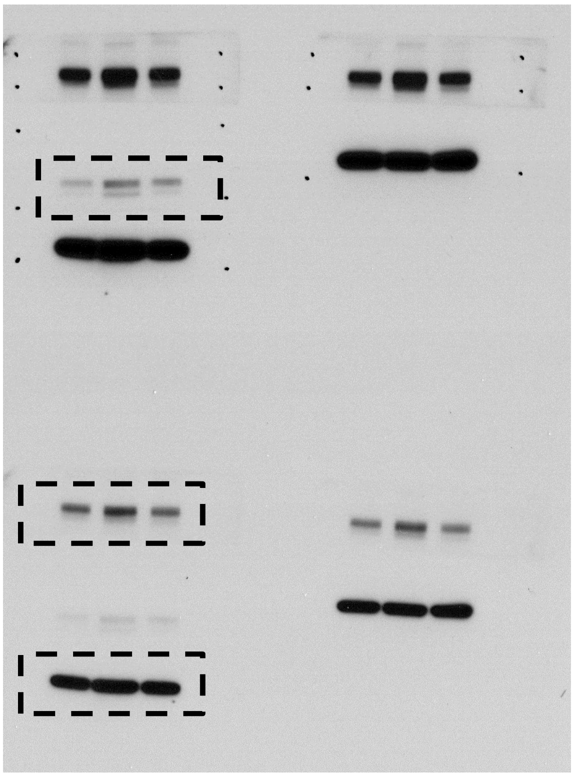

mHSC

Supplementary Material

Gel scans from the main figures

Figure 6. G

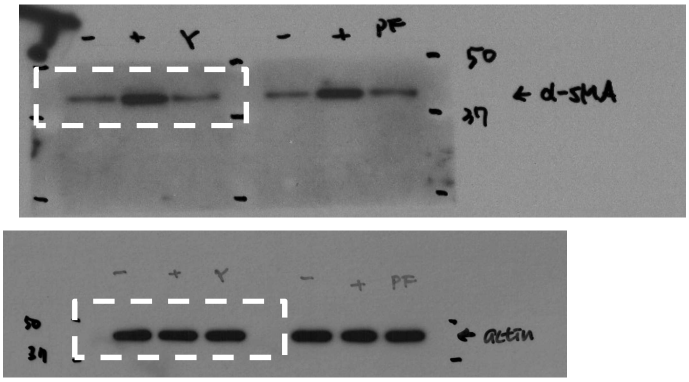

LX2

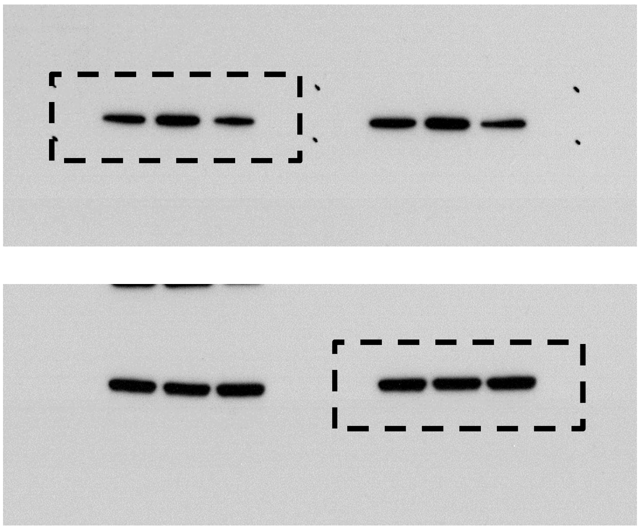

mHSC

Supplementary Material

Gel scans from the main figures

Figure 7. B

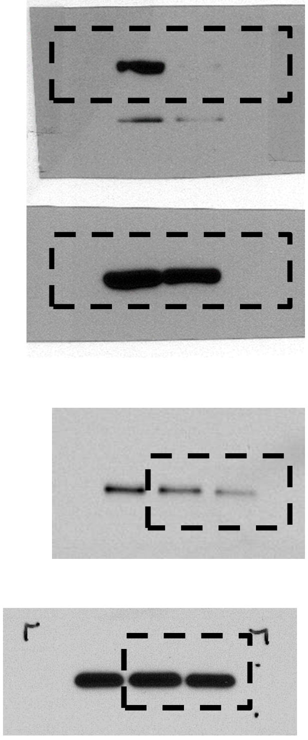

Figure 7. G

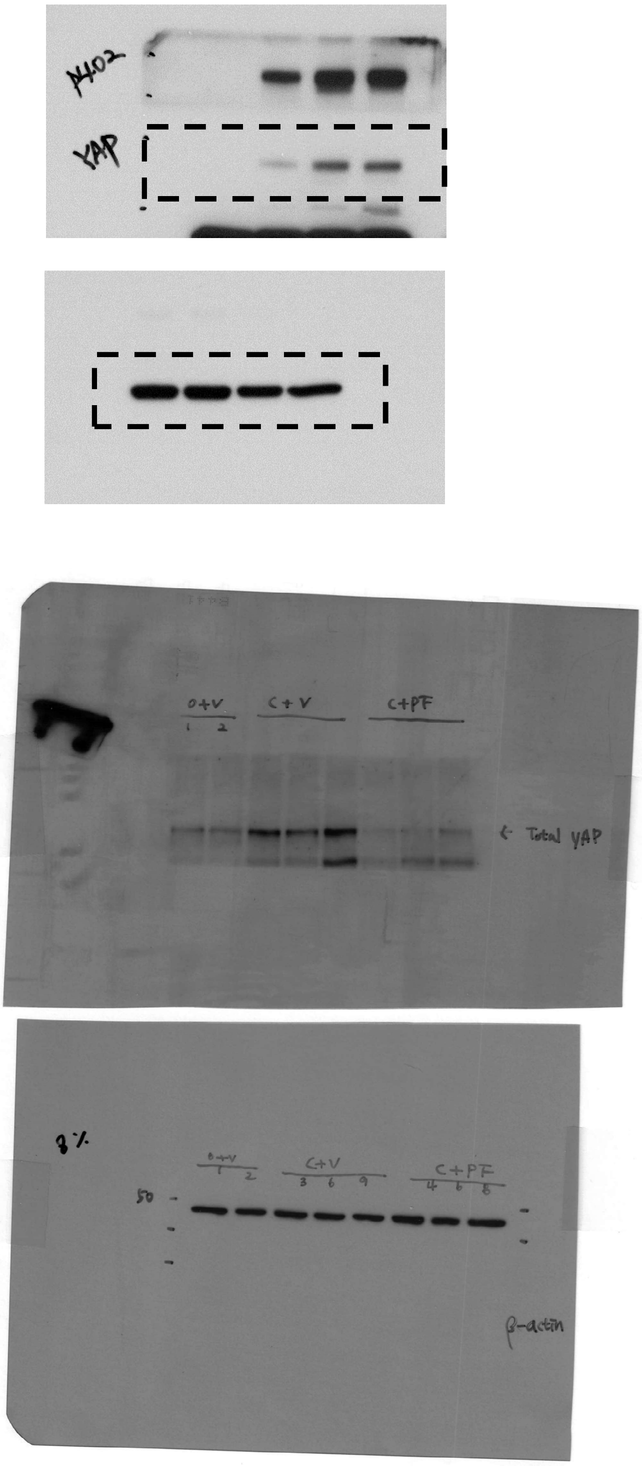

Supplement: Supplementary file 1 — Supplementary information. [file 41598_2020_78056_MOESM1_ESM.pdf]
